# An online program with individualized vs automated support for significant others of depressed individuals – study protocol of a randomized controlled trial

**DOI:** 10.1186/s12888-022-04035-6

**Published:** 2022-07-28

**Authors:** Elisabeth Schramm, Christoph Breuninger, Nadine Zehender, Ulrich Hegerl, Anne Elsner, Andy Maun, Marina Schmölz, Christiane Roick, Marlon Grodd, Erika Graf

**Affiliations:** 1 Department of Psychiatry and Psychotherapy, Medical Center–University of Freiburg, Faculty of Medicine, University of Freiburg, Freiburg, Germany; 2 Department of Psychiatry, Psychosomatic Medicine and Psychotherapy, University Hospital Frankfurt, Goethe University Frankfurt (Distinguished Professorship Funded By Dr. Senckenbergische Stiftung), Frankfurt am Main, Germany; 3 Stiftung Deutsche Depressionshilfe, Leipzig, Germany; 4 Institute of General Practice / Family Medicine, Faculty of Medicine, Medical Center–University of Freiburg, Freiburg, Germany; 5 AOK-Bundesverband, Berlin, Germany; 6 Institute of Medical Biometry and Statistics, Medical Center - University of Freiburg, Faculty of Medicine, University of Freiburg, Freiburg, Germany

**Keywords:** Major depression, Relatives, Caregiver, Depression treatment, Online program, Self-help, Randomized controlled trial

## Abstract

**Background:**

Due to budget restrictions in mental health care, non-professional caregivers are increasingly burdened with the emotional and practical care for their depressed relatives. However, informal family caregiving is mostly a stressful role with negative consequences on the physical and mental health of the caretakers to the extent that they have an elevated risk of experiencing psychiatric disorders themselves. While psychoeducation for relatives of depressed individuals showed positive results both in terms of the caretakers’ strain and the depressive symptoms of the affected person, there are major barriers to participate in presence in those programs. Digital programs might be a viable alternative. We found no empirically evaluated digital program available for informal caregivers of depressed patients.

**Methods:**

An online program for relatives of depressed individuals has been developed including four interactive modules on 1) psychoeducation, 2) how to strengthen the relationship with the depressed person, 3) how to deal with the depressive symptoms of the patient, and 4) find the right balance between caring for the depressed person and self-care. We investigate if this self-help program is more effective when used with individualized versus automated e-mail support, and if both supported conditions are more effective than treatment-as-usual (TAU in form of written information material) in terms of the risk of mental diseases in caregivers. The primary outcome is the reduction of the caregiver’s nonspecific mental distress as measured by the change of the Kessler Psychological Distress Scale score from baseline to four weeks after randomization. Caregivers (*n* = 500:500:250) will be randomized to one of the three conditions.

**Discussion:**

Psychological support for caregivers of individuals with mental disorders such as depression should be offered as part of integrated services. There is a huge potential to develop and implement interactive online approaches to support informal caregivers of patients with depression to function in their multiple roles and to help them to remain healthy.

**Trial registration:**

DRKS, DRKS00025241. Registered 5 Mai 2021.

**Supplementary Information:**

The online version contains supplementary material available at 10.1186/s12888-022-04035-6.

## Background

Depressive disorders belong to the most common mental health problems worldwide with a prevalence of 4.7% (95% CI 4.4–5.0%; [28], and are ranked as the biggest contributor to global disability [77]. Depression is associated with considerable objective and subjective stress not only for the affected person, but also for caring relatives and partners [34, 44, 52, 64, 79]. Several studies have shown that caregiving imposes a great amount of burden on the caregivers and has detrimental consequences for their mental and physical health [2, 6, 22, 73]. The *objective* care burden refers to a range of physical, financial, social, as well as time- and work-related problems. The *subjective* burden includes emotional and psychological distress such as worries, anger, guilt, frustration, sadness, and psychosomatic complaints [29, 34, 63, 79].

### Psychosocial burden of relatives and caregivers

When compared with caregivers of patients with chronic illnesses and with non-caregivers, caregivers of depressed individuals show a larger burden in terms of poor quality of life, marked impairment of productivity, and increased health resource utilization [6]. Depression puts a major strain on the partnership and the family: 84 percent of depressed individuals withdraw from social relationships during the course of a depressive disorder [71]. Half of those affected by depression experience problems in their partnership, and in 45 percent of these cases the couple ends up separated. The vast majority of significant others are concerned about the health (95%) and future (86%) of the depressed person, as well as their own future (66%; [34]. Previous studies are stressing that these strains and the care burden can be a risk factor for the development of caregivers’ own depression [9, 21, 70, 79]. The 1-year prevalence of depression is found to be more than doubled among spouses of depressed individuals in comparison to the general population [75]. Large surveys report increased symptoms of anxiety and depression in spouses of individuals with mental disorders [46] and sociodemographic factors were not sufficient to explain this concordance [16]. In addition, there is evidence suggesting an interaction between the stress experienced by relatives and the course of the patient’s depression. A greater burden on relatives is associated with an increased risk of recurrence and chronic course of the depressive disorder [45, 60].

Despite the increasingly important role of relatives as informal caregivers [40, 42] and despite the fact that they face significant care burden [34], this highly stressed group is mostly neglected by health care systems. Furthermore, caregiver overburden is frequently overlooked by clinicians [1]. A recent meta-ethnographic synthesis [63] pointed the need to offer professional support for caregivers for their mental health to prevent the development of their own depression.

### Psychoeducation and support for relatives and caregivers

Nearly one in three relatives of depressed patients report to be poorly informed about depression [71] which results in misinterpretation of depressive symptoms and in less understanding and support for the patient. Being well informed about the disorder leads to a better comprehension of the disease in both patients and relatives and to higher treatment satisfaction on the part of the patient [47]. Psychoeducational programs for relatives of depressed patients including problem-solving and coping strategies in addition to knowledge transfer, reduced the objective and subjective burden on the relatives [48, 68]. After the intervention, the percentage of relatives showing signs of anxiety and depression decreased from 50 to 9%. Furthermore, the relapse rates in the depressed patients at 9-month follow-up was only 8% in the psychoeducation compared to 50% in the control group (treatment-as-usual; [68]. A review on psychoeducation for families of depressed individuals showed positive results both in terms of the families’ strain and the depressive symptoms of the affected person [12]. In line with these findings, there is evidence from a systematic review and meta-analysis of 21 RCTs (*n* = 1589) suggesting that both psychoeducational groups as well as support groups are effective for caregivers of individuals with severe mental disorders [78].

The importance of psychoeducation for relatives and caretakers is also underlined by a recommendation in the national clinical guidelines for unipolar depression for Germany [14, 24] as well as for Australia [57]. In the NICE framework [59], informal caregivers’ needs for information and support are addressed in a dedicated guideline which is explicitly referenced in the guideline on depression [58]. However, there is a dramatic undersupply of psychoeducational offers for families of patients with depression. In a survey of all psychiatric hospitals in Germany, Austria and Switzerland, 67% of the hospitals stated that they offer psychoeducation for depressed patients and their relatives [65]. However, only 13% of the relatives participated in these face-to-face psychoeducation groups. Long distances, inflexible times, and fear of stigmatization were the main reasons given for the low rate of participation. Other studies point out more barriers for both relatives and patients, such as living in a rural area, matching provider availability with work schedules, and stigma [20, 30]. Therefore, remotely accessible interventions can be an important additional resource for patients as well as for relatives. The advantages of online compared to face-to-face interventions include anonymity, accessibility, flexibility, visually appealing and interactive interface, and cost-effectiveness [26, 55].

A systematic review and meta-analysis [67] about the impact of internet-based interventions on the mental health of caregivers living with patients with a chronic condition (mostly dementia and cardiovascular disorders) found evidence for the benefit of those programs, particularly for the outcomes of caregiver depression, anxiety, stress and distress. The forms of interventions which were most effective included information and education with or without psychological support. A more recent review [7] identified only 4 randomized and 5 nonrandomized clinical studies on online interventions for families of patients with severe mental disorders (psychosis, schizophrenia, schizoaffective, bipolar disorder, and psychotic disorder). Caregivers as well as patients showed high acceptance, good adherence and also satisfaction with the digital interventions. The review concluded that online intervention programs were superior to standard of care with respect to reducing caregivers’ symptoms, such as perceived stress and burden, and they helped to improve knowledge.

To our knowledge, there is no evidence-based online intervention currently available for non-professional caregivers of depressed patients. In one published pilot study, an E-care program for informal caregivers of depressives did not lead to a reduction in symptoms of psychological distress in the relatives [11]. An interactive online program for relatives and significant others of depressed individuals (www.familiencoach-depression.de) has been developed based on the current scientific literature at the University of Freiburg Medical Centre in cooperation with the German AOK health insurances federal association. This program’s contents are based on scientific evidence, but the program’s efficacy itself has not been evaluated so far.

### Efficacy of self-guided versus expert-guided online interventions

The efficacy of internet-based interventions in the prevention and treatment of mental disorders is evaluated positively in several reviews and meta-analyses [3, 17, 25, 43], even though there is only a small number of studies. Self-guided online-interventions have been found to be significantly less effective than expert-guided interventions [8]. It is unclear if therapeutic guidance by highly qualified coaches is more effective than automated practical and motivational guidance [3, 4]. Attempts to automate human guidance which would help to scale up preventive interventions to a broad public, are still in their infancy [49]. In the context of the present study, we aimed to develop and evaluate both an individualized message system with support provided by trained clinical psychologists, as well as an automated message system intending to keep the users motivated and engaged and thus increase the effectiveness of the online program.

### Aims

We hypothesize that: a) the online self-help program with IND or AUT reduces the nonspecific mental distress (and thus risk of mental diseases) of the caregivers more than TAU. In a comparison of the two support conditions, the individualized version will be more effective than the automated support; b) the online self-help program with individualized or automated e-mail support reduces the psychosocial burden and the subjective symptom burden of the caregivers as well as the depressive symptoms in the affected person. Furthermore, the program improves depression literacy, interaction behavior, and the well-being of the caregivers.

## Methods

### Study setting

The study takes place on the internet platform (www.gemeinsam-durch-die-depression.de). There will be neither additional phone contact nor face-to-face contact. The study is addressed to German-speaking participants in Germany. After registration on the online platform, the online intervention and the messaging system are available in the protected login area of the website.

### Trial design

The study trial is designed as a randomized, controlled open-label superiority trial with three parallel groups. The randomization will be performed as block randomization with a 2:2:1 allocation (IND:AUT:TAU). Stratification factors and further randomization details are given in additional file 1). The study protocol was compiled in accordance with the Standard Protocol Items: Recommendations for Interventional Trials (SPIRIT Checklist).

Before registration on the website, interested caregivers are provided with written information on the study and a study e-mail-address to answer any remaining questions. Informed consent is obtained from participants by actively confirming a button at the end of the informed consent and data protection summary. Afterwards, caregivers can register on the website by filling in their e-mail-address and responding to a verification e-mail. Then, the caregiver fills in a short questionnaire to check the in- and exclusion criteria (screening). If the caregiver fulfils the criteria, he/she will be included in the study. The caregiver is asked if they would like to invite the depressed person to participate as well. A personalized invitation e-mail is provided to the caregiver, who can forward it to the depressed person. The invitation contains a link to a study information and informed consent page tailored to depressed participants. Interested depressed participants can thus register themselves with an individual account that is linked to the corresponding relative.

**Fig. 1:**
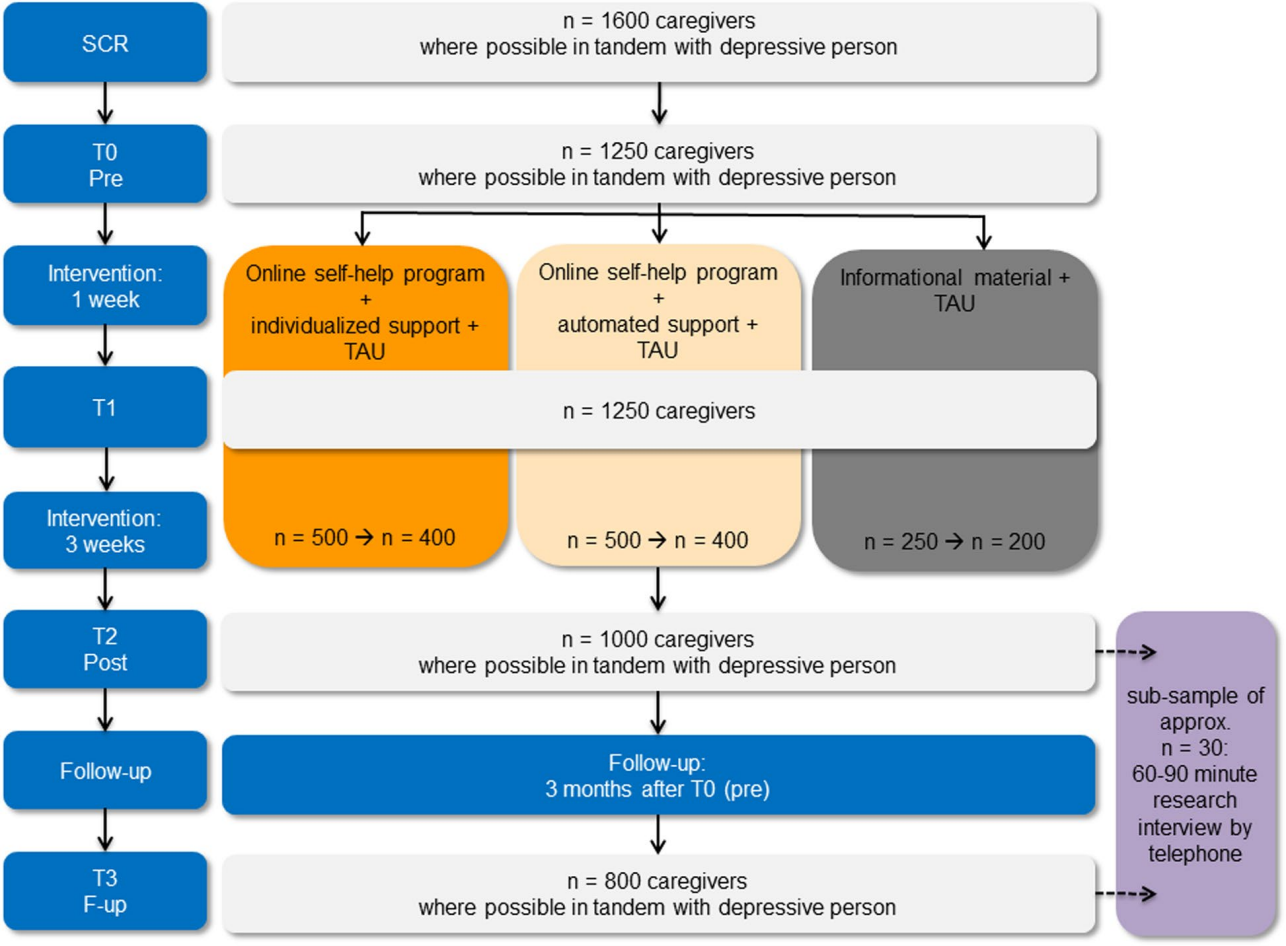
Participant flow and randomization

After completing the pre-intervention questionnaires (T0), the caregiver will be randomized to one of the three interventions. One week after randomization (T1), the caregiver will be asked to complete a short questionnaire concerning the primary outcome, his/her psychological distress (K-10). Following the four-weeks intervention period, the caregivers (and depressed relatives, if available) fill in the questionnaires again (T2). At this time, there will be additional questionnaires for those caregivers who were randomized to one of the active interventions, asking for the acceptance, adherence to and usage of the online program. Adverse events that might have occurred during the intervention are ascertained from all caregivers. Three months after T0 (T3), the caregivers (and depressed relatives, if available) complete the psychological questionnaires again und the caregivers will be asked again for adverse events during the past eight weeks. To improve data completeness, the participants will get repeated e-mail reminders if they don’t complete the questionnaires at T1, T2 or T3. For completing all measurements, the caregivers get a compensation in the form of a 30€ voucher (10€ for each measurement point; the vouchers can be used at a large number of online and local stores and services; funding for the vouchers is included in the study grant). The study flowchart is shown in Fig. 1.

For risk assessment, (serious) adverse events ((S)AE) were defined ahead of the study and recorded at T2 and T3 and during the psychological support. In case of a SAE there is an emergency button on the website of the online program, where the participant can find information and contacts of emergencies services and will be instructed on how to contact them.

### Sample size

In a sequential closed test procedure, confirmatory pairwise comparisons of randomized arms using two-group t-tests at two-sided significance level α = 5% will continue until the first non-significant result. They will be implemented via calculation of confidence intervals. The first and second statistical test will compare the online self-help program interventions with control to reject equalities IND = TAU and AUT = TAU of mean pre-to-post-(T2-T0) K-10 changes. With *n* = 400:400:200 (IND:AUT:TAU) non-missing primary outcomes, the statistical power for these tests is 1-β≈99% given a medium effect size (Cohen’s d) of d = 0.5, and 93% if d = 0.3. The third test comparing both online self-help interventions to reject IND = AUT has a power of 81%, assuming a small effect size of d = 0.2 between the support variants. Confirmatory testing will stop after this comparison. To account for an expected 20% rate of missing outcomes at T2, *n* = 500:500:250 caregivers will be randomized (see additional file 1 for further details).

### Inclusion and exclusion criteria

Caregivers can participate in the trial alone or together with their depressed relative, significant other or close friend. In either case, some information on the depressed person is obtained from the caregivers, and both must meet the in- and exclusion criteria shown in Table 1. Both caregivers and depressed persons have to be 18 years of age or older.

**Table 1:** In- and exclusion criteria for caregivers participating in the program and for depressed relatives optionally participating in assessments

	**concerning the caregiver**	**concerning the depressed person**
inclusion criteria	• age 18 years and older • sufficient German language skills • e-mail account and access to the internet • informed consent • caring for a depressed person	• age 18 years and older • sufficient German language skills • e-mail account and access to the internet • informed consent • primary diagnosis of unipolar depressive disorder OR primarily depressive symptoms (according to caregiver)
exclusion criteria	• suffering from any current mental disorder	• *primary* diagnosis of any other mental disorder (e.g., bipolar disorder, schizophrenia)

### Recruitment

Participants will be recruited throughout Germany. As the majority of depressed persons are treated by general practitioners [36], general practitioner practices as well as specialist practices for psychiatry and psychotherapy will be informed and sensitized to the role of caregivers in the treatment of depression. Clinics for psychiatry and psychotherapy will be included in the recruitment strategy as well. We will provide information material for patients and caregivers (posters, flyers) to practices and clinics. More caregivers will be addressed through online forums, self-help groups for caregivers, self-help contact bureaus, crisis intervention centers and caregivers’ associations. Furthermore, information on study participation will be disseminated through the network of 85 regional alliances against depression (Bündnisse gegen Depression) and a patient congress, both organized by the German Depression Foundation (Stiftung Deutsche Depressionshilfe). Also, their various channels are used in order to specifically reach relatives of people with depression, among others through the highly frequented informational website as well as the wide-reaching social media presentation of the German Depression Foundation. The public will also be addressed through the magazines and internet pages of the cooperating health insurance AOK and via advertisements in search engines, social platforms, and other media.

### Intervention

Only the caregiver will receive one of the three interventions.

The online self-help program consists of four interactive, independent modules. In these modules, caregivers are (1) instructed on how to deal with their relatives’ depressive symptoms, (2) informed about depression as a mental illness including suicidality and specific treatment options, (3) advised on how to strengthen the relationship with the depressed person, and (4) provided instructions and exercises on finding the right balance between caring for the depressed person and self-care. Completing each module will take approximately 1.5 to 2.5 h per module including homework exercises.

During the study period of four weeks, the online self-help program will be offered in the following three individually randomized variants:

1. *Online self-help program with individual support*: The individual e-mail-support will be implemented by trained psychologists under regular supervision with experienced psychotherapists. The psychologist contacts the caregivers three time per week via a secure message system to direct the caregiver to individually relevant contents, give additional information about the modules (e.g. by relating the content to the personal situation), answer individual questions and strengthen the motivation of the caregivers in dealing with obstacles.
2. *Online self-help program with automated support:* The fully automated message system includes regular standardized motivational e-mails, reminders with encouraging messages, and feedback on completed modules, tailored to each participant’s activities and progress in the online program. In the development of the automated support system, a focus group consisting of experts in e-mental-health and psychoeducation for relatives was conducted, and caregiver experiences were collected in qualitative interviews in a pilot study.
3. *Treatment as usual (TAU) control condition with written information material*: In the TAU condition, the caregivers receive a digital version of the patient information leaflet „Depression – Angehörige und Freunde“, which was developed by the ÄZQ based on patient guidelines of unipolar depression (Ärztliches Zentrum für Qualität in der Medizin (ÄZQ), [5]). On two pages, this document presents concise information on living with the situation, supporting the depressed person, managing crises and caring for oneself. After the study period of three months, the caregivers of the TAU control condition can also use the online self-help program with automated support. Participants in all groups are free to use medical or psychological services as usual, but support services specifically for relatives are very rarely available.

### Primary outcome measure

#### Kessler PSYCHOLOGICAL Distress Scale (K-10)

The K10 scale [50],German version: [37] assesses non-specific psychological distress during the past 30 days. It includes ten items on a five-point Likert scale and five additional items. The answer options for the first ten items range from one (none of the time) to five (all of the time). Questions begin with “During the last 30 days, about how often did you …” and continue with (1) … feel depressed (2) … feel tired out for no good reason? (3) … feel nervous?” The maximum score is 50, indicating high psychological distress. Ten, the minimum score, indicates the absence of psychological distress. The additional items relate to the frequency of psychological distress in the past 30 days as compared to other times, the performance, the frequency of medical visits and the attribution of physical diseases on the perceived distress.

Giesinger et al. [37] report good internal consistency of the scale (Cronbachs Alpha ranging from 0.80 – 0.90). To measure convergent validity, satisfactory correlations with related scales could be found (correlation with the State-Anxiety-Scale of the STAI [53] *r* = 0.68 and with the scale GSI of the BSI [33] *r* = 0.71).

The outcome measures and assessment times are presented in Table 2.

**Table 2:**
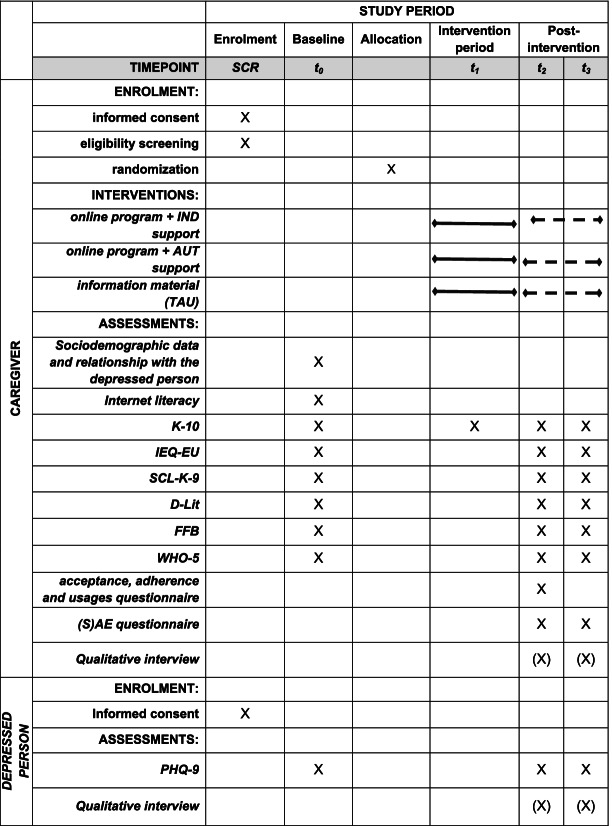
Outcome measures and assessment times

Participants can continue using the assigned intervention during the post-intervention period, but participants randomized to a condition including the online program cannot receive or exchange any further messages, indicated by the dashed lines

### Secondary outcome measures

#### The involvement evaluation questionnaire (IEQ_EU)

The IEQ-EU (German version: [10, 66, 72] assesses the burden on caregivers of mentally ill people in the past four weeks. The IEQ-EU consists of 31 items, which are assessed on a five-point Likert scale ranging from 0 (never) to 4 (always). Of the 31 items, 27 are grouped into four subscales, namely tension, supervision, worrying, and urging. The internal consistency (Cronbachs Alpha) of the subscales is reported as moderate or good, ranging from 0.71- 0.88, for the German version [10].

#### Symptom checklist 9-item short version (SCL-K-9)

The psychological burden on caregivers of mentally ill people is evaluated by the German version of the SCL-K-9 [51] a shortened version of the Symptom Checklist 90-R [23, 32]. By using nine items on a five-point Likert scale ranging from 0 (not at all) to 4 (very severe), different psychological symptoms are assessed. Cronbach’s Alpha is reported as α = 0.87, indicating good reliability of the SCL-K-9 [61].

#### Depression literacy test (D-Lit)

The literacy concerning depression is rated using the German version of the Depression Literacy Test (D-Lit) (German version: [35, 39]. The D-Lit is a 35-item self-report questionnaire, assessing general information about depressive disorders, for example symptoms, impairments, and treatments. Response options are “true”, “false” and “don’t know”, with one point given for each correct answer. Reliability of the scale is reported as moderate with a Cronbach’s Alpha of 0.75 [35].

#### Family Questionnaire (FQ)

The level of expressed emotion (EE) is measured by the German version of the Family Questionnaire (FQ) [74], a 20-item self-report questionnaire. On a four-point Likert scale, ranging from “never” to “very often,” caregivers of patients evaluate the frequency of specific reactions towards the patient. Internal consistency was excellent for the subscale “criticism” (Cronbach’s Alpha of 0.90–0.92) and good for the subscale “emotional over-involvement” (Cronbach’s Alpha ranging of 0.79–0.82).

#### World health organization well-being index (WHO-5)

The subjective psychological well-being is evaluated by the German version of the 5-item WHO-5 (World Health Organization. Regional Office for Europe, [76]). Response options are coded on a six point scale (0 = never; 5 = all of the time). The internal consistency of the German version of the WHO-5 is excellent (Cronbach’s Alpha = 0.92; [13].

#### Patient health questionnaire (PHQ-9)

If the depressed person participates in the study as well, the person has to fill in the PHQ-9 at T0, T2 and T3. The PHQ-9 (German version: [56, 69] measures depressive symptoms. Subjects indicate for each of the nine items on a four-point scale, ranging from 0 (never) to 3 (nearly every day), whether the symptom has bothered them during the previous 2 weeks. The PHQ-9 showed excellent criterion validity (medical patients: sensitivity, 95%; specificity, 86%) and good internal consistency (Cronbach’s Alpha = 0.88; [38].

#### Usage data of the online program

The online program will collect the following usage data for each participant: individual progress in each of the four modules, number of logins and time spent in the online program each week. In the condition with individualized support, the frequency of correspondence with the psychologist will be stored as well. For content analysis, anonymized correspondence between the caregivers and support psychologists is stored. At T2 there will be an additional questionnaire for caregivers asking for the acceptance, adherence and usage of the online program. For the adherence assessment, participants are asked if they have used other support or self-help programs during the study and the usage data will be analyzed.

#### Qualitative interviews

To gather process information, individual participants (caregivers) will be selected based on theoretical sampling and interviewed by telephone for approximately 45–60 min. Trained psychological personnel using a semi-structured interview guideline conducts the interviews. The interview questions were developed for a pilot study and adapted for this study (additional file 2). The contents of the interviews with caregivers focus on the experiences with the online self-help program, the support conditions, everyday life experiences and the interaction with the depressed person as well as potential changes brought about by the intervention. The content of the interviews is recorded in the form of audio recordings and transcribed for further analysis.

### Statistical methods

In the primary intention-to-treat analysis, all randomized caregivers will be analyzed in the assigned treatment arms, irrespective of treatment adherence or discontinuation. The effects of allocation to IND, AUT and TAU with respect to the change in K-10 score from baseline to four weeks after randomization will be estimated and tested in a linear regression model. The model will include randomized treatment (IND, AUT, TAU), K-10 baseline scores, age (18–40, 41–65, ≥ 66 years), caregiver’s relation with depressed person (parent, child, partner, other) and sex as independent variables. Since very few caregivers of diverse sex are expected, they will be assigned alternatingly to the groups of females and males in the primary analysis of intervention effects. Following a sequential closed testing procedure to ensure a multiple type I error rate of 5%, confirmatory comparisons of randomized treatment arms will proceed until the first occurrence of a non-significant result at the nominal 5% level, followed by descriptive reporting of all subsequent analyses. Tests of equality of means of two treatment arms will be based on the two-sided 95% confidence interval for the difference in mean change from baseline estimated from the linear regression model. Treatment arms will be compared in the following order: IND versus TAU, AUT versus TAU, IND versus AUT. Missing values will be replaced by multiple imputation using baseline data and post-baseline information according to a Treatment Policy Strategy as described by Polverejan and Dragalin [62] and Guizzaro et al. [41].

Although it is hypothesized and subjected to confirmatory testing that IND is superior to AUT, equality of the less costly intervention AUT compared to the more costly intervention IND is also deemed possible. In a secondary descriptive analysis, demonstration of non-inferiority of AUT compared to IND will also be attempted. If the lower limit of the two-sided 95%-confidence interval for the difference of mean K-10 changes from baseline (IND minus AUT) is greater than -0.62 points (Cohen’s *d* > -0.1), this will be interpreted as non-inferiority (see additional file 1 for derivation of the margin and statistical power).

The primary evaluation will be complemented by sensitivity analyses with respect to the missing data mechanism and the effect of adherence. In order to assess the consistency of the treatment effect in relevant subgroups (EMA-CHMP, [27]), exploratory analyses will be performed using all randomization stratification variables (i.e. age, sex, relation with depressed person, pre-intervention (T0) K-10 score) as well as internet literacy. Here, caregivers of diverse sex will be considered separately from males and females. Further analyses will explore possible moderators and mediators.

Secondary outcomes at T2, four weeks after randomization, and T3, three months after randomization, will be evaluated in a linear model per outcome scale, with a compound symmetry covariance matrix to account for correlation between T2- and T3-outcomes of the same caregiver or depressed person. Independent variables will be those of the main primary analysis, plus time point (T2, T3). Subgroup analyses, moderator and mediator analyses will be performed in a similar fashion as for the primary outcome. Furthermore, the results seen in the current trial will be compared to historical controls of face-to-face psychoeducation groups for caregivers of depressive in-patients from the multicenter SCHILD study [29, 31].

Further details of the analysis strategy are described in the full study protocol (see additional file 1) or will be pre-specified in a statistical analysis plan, to be completed during a blinded review of trial data. Moderator and mediator analyses will be pre-specified and conducted separately from the main project as part of PhD theses.

### Qualitative methods

Based on the interview transcripts, a qualitative content analysis is carried out. The focus of the qualitative analysis is on the subjective perception of the interviewed person. Depending on the research question, an appropriate method within the grounded theory framework is selected. Our research group is experienced in both constructivist grounded theory analyses [18, 19] and core sentence (“Kernsatz”) analyses [15, 54]. For the messages between participants and psychologist from the individual support condition, an appropriate form of qualitative or quantitative content analysis is chosen for each research question.

## Discussion

This protocol describes an RCT to evaluate the effects of an online self-help program for caregivers of depressed persons with individualized (IND) or automated (AUT) support in comparison with a TAU condition. The self-help program consists of four interactive, independent modules addressing information on depression, how to handle depressive symptoms of the affected person, how to strengthen the relationship with the depressed person, and the balance between self-care and caring for others. The intervention aims at reducing the caregiver’s mental distress as a risk factor for mental disease. A diminished burden in caregivers is expected to also have a positive influence on the course of the depressive disorder in the affected person.

Particularly, relatives and significant others of depressed individuals have an essential role in providing care to the patients, which may impose significant burden on them. However, relatives, significant others, and other caregivers of depressed individuals are a group mostly neglected by health care systems, even though supporting them may improve well-being and health outcomes of both caregivers and patients and despite the fact that national and international clinical guidelines on depression recommend psychoeducation and support for this group [24, 57, 58]. A positive evaluation of the online intervention could be of significant public health and economic relevance by scaling up preventive online interventions to a broad public of affected individuals and their caregivers.

## Supplementary Information

**Additional file 1.**

**Additional file 2.** Interview questions.

## Data Availability

Not applicable.

## Abbreviations

IND: Online self-help program with individualized support
AUT: Online self-help program with automated support
TAU: Treatment as usual (written informational material)
RCT: Randomized controlled trial
(S)AE: (Severe) Adverse events
