# Supplementary material for: An online program with individualized vs automated support for significant others of depressed individuals – study protocol of a randomized controlled trial
Source: BMC Psychiatry. 2022 Jul 28;22:511. doi: 10.1186/s12888-022-04035-6 (PMC9331487; doi:10.1186/s12888-022-04035-6)
Supplement: Supplementary file 1 — Additional file 1. [file 12888_2022_4035_MOESM1_ESM.pdf]

CLINICAL TRIAL PROTOCOL

**Facing Depression Together –
Evaluation of an online self-help program with individualized vs automated supportive message system for relatives, significant others, and caregivers of depressed persons**

Version 2.0, October 2021

<u>Principal Investigator</u>
Prof. Dr. phil. Elisabeth Schramm
University Medical Center Freiburg

<u>Sponsor</u>
University Medical Center Freiburg

1 **Abbreviations** **4**
2 **Administrative information** **5**
2.1 Roles and responsibilities 5
2.2 Funding 5
3 **Introduction** **5**
3.1 Background and rationale 5
3.2 Objectives and estimand 9
3.3 Hypothesis 10
3.4 Trial design 10
4 **Methods: Participants, interventions and outcomes** **10**
4.1 Study setting 10
4.2 Eligibility criteria 11
4.2.1 Criteria for participation of caregivers 11
4.2.2 Criteria for participation of depressed person 11
4.3 Interventions 11
4.4 Outcomes 14
4.5 Participant timeline 14
4.6 Sample Size 16
4.7 Recruitment 17
5 **Methods: Assignment of interventions** **18**
5.1 Allocation 18
5.1.1 Sequence generation 18
5.1.2 Allocation concealment mechanism 19
5.1.3 Implementation 19
5.2 Blinding (masking) 19
6 **Methods: Date collection, management and analysis** **19**
6.1 Data collection methods 19
6.1.1 General Data 19
6.1.2 Psychological variables 21
6.1.3 Usage Data of the online program 23
6.1.4 Data collection quality 23
6.1.5 Collection of qualitative interview data 24
6.2 Data management 25
6.2.1 Storage of data 25
6.2.2 Data Transfer 26

6.2.3 Data protection/Data security 26
6.2.4 Data integrity 28
6.3 Statistical methods 28
6.3.1 Biostatistical planning and analysis 28
6.3.2 Main analysis of primary outcome 29
6.3.3 Sensitivity analysis of primary outcome 30
6.3.4 Supplementary analysis of primary outcome 31
6.3.5 Analysis of secondary outcomes 31
6.3.6 Further supplementary analyses 31
6.4 Qualitative methods 32
7 Methods: Monitoring 32
7.1 Data Monitoring 32
7.2 Harms 32
7.3 Auditing 33
8 Ethics and dissemination 34
8.1 Research ethics approval 34
8.2 Protocol amendments 35
8.3 Consent or assent 35
8.4 Confidentiality 35
8.5 Declaration of interests 36
8.6 Access to data 36
8.7 Ancillary and post-trial care 36
8.8 Dissemination policy 37
9 References 37

1 Abbreviations

AUT: Online self-help program with automated support

IND: Online self-help program with individual support

TAU: Treatment as usual control condition (conventional treatment under conditions of routine care); in this case written information material for relatives of persons with depression

2 Administrative information

2.1 Roles and responsibilities

- Principal Investigator: Prof. Dr. phil. Elisabeth Schramm, Department of Psychiatry and Psychotherapy, Medical Center–University of Freiburg, Faculty of Medicine, University of Freiburg, Freiburg, Germany
- Study coordination: Dipl.-Psych. Christoph Breuninger & M. Sc. Psych. Nadine Zehender, Department of Psychiatry and Psychotherapy, Medical Center–University of Freiburg, Faculty of Medicine, University of Freiburg, Freiburg, Germany
- Statistical Analysis: Dr. rer. nat. Erika Graf, Institute of Medical Biometry and Statistics, Faculty of Medicine, University Medical Center Freiburg, University of Freiburg, Freiburg, Germany
- Recruitment Support: Prof. Dr. med. Ulrich Hegerl, Leipzig, Vorsitzender der Stiftung Deutsche Depressionshilfe
- Recruitment Support: Prof. Dr. med. Andy Maun & Prof. Dr. med. Wilhelm Niebling, Division of General Practice, Medical Center – University of Freiburg, Faculty of Medicine, University of Freiburg, Freiburg, Germany
- Technical implementation of the E-Mail support and online platform: Prof. Gábor Kovács; Berlin; Prodekan Fachbereich Design, Onlinekonzeption

2.2 Funding

Financing for this trial has been secured by a grant from the “Innovationsfonds zur Förderung von Versorgungsforschung (§ 92a Abs. 2 Satz 1 SGB V)” with the reference 01VSF19054.

3 Introduction

3.1 Background and rationale

Depressive disorders are among the most common mental disorders globally, and are ranked by the WHO as the single largest contributor to global disability (World Health Organization, 2017). In Germany, 4.9 million adults are estimated to suffer from unipolar depression annually (Jacobi et al., 2014). During the course of their lives, a majority of Germans are affected by depression either directly (23%) or indirectly as relatives or close friends (37%, Deutsche Depressionshilfe, 2018). Depressive

disorders are a leading cause of burden of disease (years lived with disability), both globally and especially in developed countries like Germany (Vos et al., 2012). Societal costs are also very high, with an estimated 210,000 work years lost annually in Germany (Robert Koch-Institut, 2018). Direct treatment costs in Germany are estimated at 4.6 billion Euro per year (König, Luppa, & Riedel-Heller, 2010), most of which is attributable to inpatient treatment and medication. Apart from pharmacotherapy, psychotherapeutic treatments are considered first-line treatments for depressive disorders (Meister et al., 2018) as recognized in national guidelines (DGPPN, BÄK, KBV, & AWMF (Hrsg.) für die Leitliniengruppe Unipolare Depression, 2015).

Relatives, significant others and other caregivers of depressed persons are a group mostly neglected by current health care systems, despite the fact that they face increased pressures on in the form of objective and subjective burdens and caring responsibilities for the patients as a result of deinstitutionalization of mental health services (Franz, Meyer, & Gallhofer, 2003). Depression puts a great strain on partnership and family: 84 percent of those affected withdraw from social relationships during a depression, according to a recent representative survey by the German Foundation for Depression (Deutsche Depressionshilfe, 2018). Half of those affected by depression experience problems in their partnership, and in 45 percent of these cases the couple ends up separated. The depressive disorder is associated with considerable subjective and objective stress, even for healthy relatives and partners (Bischkopf, Wittmund, & Angermeyer, 2002; Franz et al., 2003): the vast majority of relatives and partners are concerned about the health (95%) and future (86%) of the depressed person, as well as their own future (66%; Franz et al., 2003). The majority suffers from negative feelings such as guilt, shame or fear of the future and experience physical and psychosomatic complaints. (Frank, Hasenmüller, et al., 2015; Franz et al., 2003). As a result, the relatives' ability to cope with everyday life, their relationships and leisure activities are often impaired. These strains also manifest in the caregivers‘ risk of developing a depressive disorder themselves. The 1-year prevalence of depression is found to be more than doubled among caregivers of depressed individuals in comparison to the general population (Butterworth & Rodgers, 2006; Idstad, Ask, & Tambs, 2010; Wittmund, Wilms, Mory, & Angermeyer, 2002). An international review (Steele, Maruyama, & Galynker, 2010) has shown that relatives of people with bipolar disorder, where depressive episodes usually predominate, have

significantly higher rates of depression and other mental illnesses, combined with an increased health care utilization. Reducing this risk should be a central goal of health policy, because a first depressive episode is followed by a recurrent depressive disorder in at least 50% of cases, and thus leads to further episodes, with the afore-mentioned consequences for those affected and for society. Also, there is evidence suggesting an interaction between the stress experienced by relatives and the course of depression in the patients. A greater burden on relatives significantly increases the risk of recurrences and chronic courses of the disease (Hölzel, Härter, Reese, & Kriston, 2011; Perlick, Rosenheck, Clarkin, Raue, & Sirey, 2001).

Almost one in three relatives say that they are poorly informed about depression (Deutsche Depressionshilfe, 2018). However, lack of knowledge among relatives results in less understanding for the patient, less support and misinterpretation of depressive symptoms such as social withdrawal and reduced emotions. On the other hand, a review (Jacob & Bengel, 2000) concluded that specific information for patients and relatives about the disease depression lead to a better understanding of the disease and a higher treatment satisfaction on the part of the patient. Psychoeducational offers for relatives, which include problem-solving and coping strategies in addition to pure knowledge transfer, can reduce the objective and subjective burden on relatives (Katsuki et al., 2011; Shimazu et al., 2011). After the intervention, the percentage of the relatives showing signs of anxiety and depression could be reduced from 50% to 9%. There is also evidence suggesting that recurrence of depression has been reduced after the relatives' participation in psychoeducational courses (Shimazu et al., 2011): The authors report the recurrence rate at 9-month follow-up to be only 9% in the psychoeducation group compared to 50% in the randomized treatment-as-usual control group. In line with this, a more recent paper reviewing 10 studies on psychoeducation for relatives of depressed individuals showed predominantly positive results both in terms of the relatives' strain and the depressive symptoms of the affected (Brady, Kangas, & McGill, 2017). A multi-center study (SCHILD) on group psychoeducation relatives of persons in inpatient depression treatment has just been completed in Germany, but the results are not published yet (Frank, Wilk, et al., 2015).

The importance of psychoeducation for relatives is also underlined by a recommendation in the current S3 guidelines/NVL "Unipolar Depression" (DGPPN et al., 2015). Despite the importance of such services for relatives, there is a dramatic

undersupply. In a survey of all psychiatric hospitals in Germany, Austria and Switzerland, only about 70% of the hospitals stated that they offered psychoeducation courses for relatives of depressive patients at all (Rummel-Kluge, Kluge, & Kissling, 2015). Moreover, only 13% of the relatives in these clinics took part in these offers. The authors discussed possible reasons for this and mentioned inflexible times of the offers, far travel to reach the clinic, and the fear of stigmatization associated with a visit to the clinic. Frank and colleges (2014) reported similar results from a survey of German acute care hospitals: Only one in three clinics offered groups for relatives of depressive patients, and only one in five relatives participated in these groups (Frank et al., 2014). Hence, only about 5-10% of relatives of depressive patients undergoing inpatient treatment are reached.

In the outpatient setting, the situation of relatives of depressive patients is even worse, as offers for relatives are not included in psychotherapy guidelines. While psychiatric specialist can provide services to relatives that are reimbursed by German public health insurances, this option is hardly used due to organizational obstacles. As only 21% of the first-depressive patients receive an inpatient treatment (Gerste & Roick, 2014), the vast majority of relatives of depressive patients do not have access to psychoeducational services provided by specialists in routine care. Due to this problematic supply situation, self-help-offers are an important additional resource for patients as well as relatives. In this area, digital services have become increasingly important throughout Germany and internationally (Deutsche Depressionshilfe, 2017). For these reasons, an online self-help program for relatives and close friends of persons with depression was developed (www.familiencoach-depression.de). The program was developed based on the current scientific literature by Prof. Schramm's working group at the University of Freiburg Medical Center in cooperation with the German AOK health insurances federal association, with the help of an expert advisory council, and with the involvement of focus groups of depressed individuals and relatives of depressed persons. The program is available freely and free of charge since 9/2018. It is met with great interest and acceptance among users as well as experts (a reference will be included in the new S3 guidelines for unipolar depression). However, the family coach depression is only available as an unaccompanied online program so far.

The efficacy of online-interventions in the prevention of mental disorders is evaluated positively in a recent review of meta-analyses (Ebert et al., 2018), even

though the authors note a comparatively small number of studies. Purely informative online-interventions are found to be significantly less effective than guided interventions (Baumeister et al., 2014). At the same time, it is unclear if therapeutic guidance by highly qualified coaches is more effective than purely practical and motivational guidance (Andersson & Titov, 2014). Attempts to automate human guidance, which would help to economically disseminate preventive interventions to a broad public, are still in their infancy (Kelders, Bohlmeijer, Pots, & van Gemert-Pijnen, 2015). Therefore, in the context of the present study we aimed to develop and evaluate both an individualized message system with support provided by trained psychologists, as well as an automated message system aimed at keeping the users motivated and engaged and thus increase the effectiveness of the measure.

3.2 Objectives and estimand

The objective of the research project is to increase the efficacy of an online self-help program for caregivers[1] of depressed persons by developing and implementing individualized vs automated support via secure e-mail messages. Within this evaluation study the following primary research question will be addressed: Which impact has the four-week usage of the online self-help program with individualized or automated e-mail support vs treatment as usual on the risk of mental diseases in caregivers? In order to achieve this, we strive to determine if a treatment policy assigning caregivers of depressed persons to an individualised or automated online self-help intervention reduces the caregiver's unspecific mental distress compared to assignment to treatment as usual (TAU), as measured by the change of the K-10 score from baseline to four weeks after randomisation (post-measurement minus baseline). The change in mental distress is considered relevant regardless of whether or not the caregiver adheres to or discontinues the assigned treatment. Treatment arms should be compared with respect to the difference in means of the change in K-10 score.

---

[1] The self-help program evaluated in this study addresses relatives as well as close friends, colleagues, roommates etc., who give support to a depressed person and are affected by the depressive disorder. In the following, they will be referred to summarily as caregivers.

3.3 Hypothesis

Primary hypotheses: The online self-help program with individualized (IND) or automated (AUT) e-mail support reduces the unspecific mental distress of the caregivers (as an indicator for the risk of mental diseases) as compared to TAU. In a comparison of the two kinds of support, the individualized support will be more effective than the automated support.

Secondary hypotheses and goals:

- The online self-help program with individualized or automated e-mail support reduces the psychosocial burden and the subjective symptom burden of the caregivers as well as the depressive symptoms in the depressed person. It improves depression literacy, interaction behaviour and the well-being of the caregivers.
- Assessing the acceptance and usage of the online self-help program
- Identification of moderators and mediator variables
- Qualitative evaluation of (a) the caregivers' experiences with the online self-help program, the support conditions, everyday life experiences and the interaction with the depressed person as well as potential changes brought about by the intervention, and (b) the depressed persons' experiences of potential changes in the caregivers behavior and their interactions.

3.4 Trial design

The study trial is designed as a randomized, controlled open-label superiority trial with three parallel groups. The randomization will be performed as block randomization with a 2:2:1 allocation (IND:AUT:TAU).

4 Methods: Participants, interventions and outcomes

4.1 Study setting

The study takes place only on the internet platform (www.gemeinsam-durch-die-depression.de). There will be neither additional phone contact nor face-to-face contact. The study is addressed to German-speaking participants in Germany. After registration on the online platform, the online intervention and the messaging system are available in the protected login area of the website.

4.2 Eligibility criteria

4.2.1 Criteria for participation of caregivers

Caregivers can participate in the trial alone or together with their depressed relative, significant other or close friend. If only the caregiver participates in the study, the participant has to provide information on themselves and the depressed person they care for, and both must meet the following in- and exclusion criteria:

Inclusion criteria concerning caregiver:

- age 18 years and older
- sufficient German language skills
- e-mail account and access to the internet
- informed consent
- caring for a depressed person, who
  - is of age 18 years or older
  - has a primary diagnosis of unipolar depressive disorder OR primarily depressive symptoms (according to caregiver)
  - has no primary diagnosis of any other mental disorder (e. g., bipolar disorder, schizophrenia).

Exclusion criteria concerning caregiver:

- suffering from any mental disorder

4.2.2 Criteria for participation of depressed person

Additionally, the depressed person can participate in the study to provide direct information on their symptoms, but only together with the caregiver. In that case, the depressed person has to provide information on and meet the following criteria:

Inclusion criteria concerning depressed person:

- sufficient German language skills
- e-mail account and access to the internet
- separate informed consent

4.3 Interventions

Only the caregiver will receive one of the three interventions. If the depressed person participates as well, he/she only fills in a short questionnaire at each measurement point (and is able to provide additional feedback on the study if desired).

The online self-help program consists of four interactive, independently usable modules (Figure 1). It instructs caregivers on how to deal with common depressive

symptoms (module „Depression und Alltag”). Furthermore, it informs about depression as a mental illness, specific treatment options, critical situations and suicidal tendencies (module „Was muss ich wissen? “) and advises on how to strengthen the relationship between the depressed person and the caregiver (module „Beziehung stärken“). It provides instructions and exercises on how to find the right balance between caring for the depressed person and self-care (module „Selbstfürsorge “). Completing each module will take approximately 1.5 to 2.5 hours per module including homework exercises.

Fig. 1 Structure und modules of the online self-help program

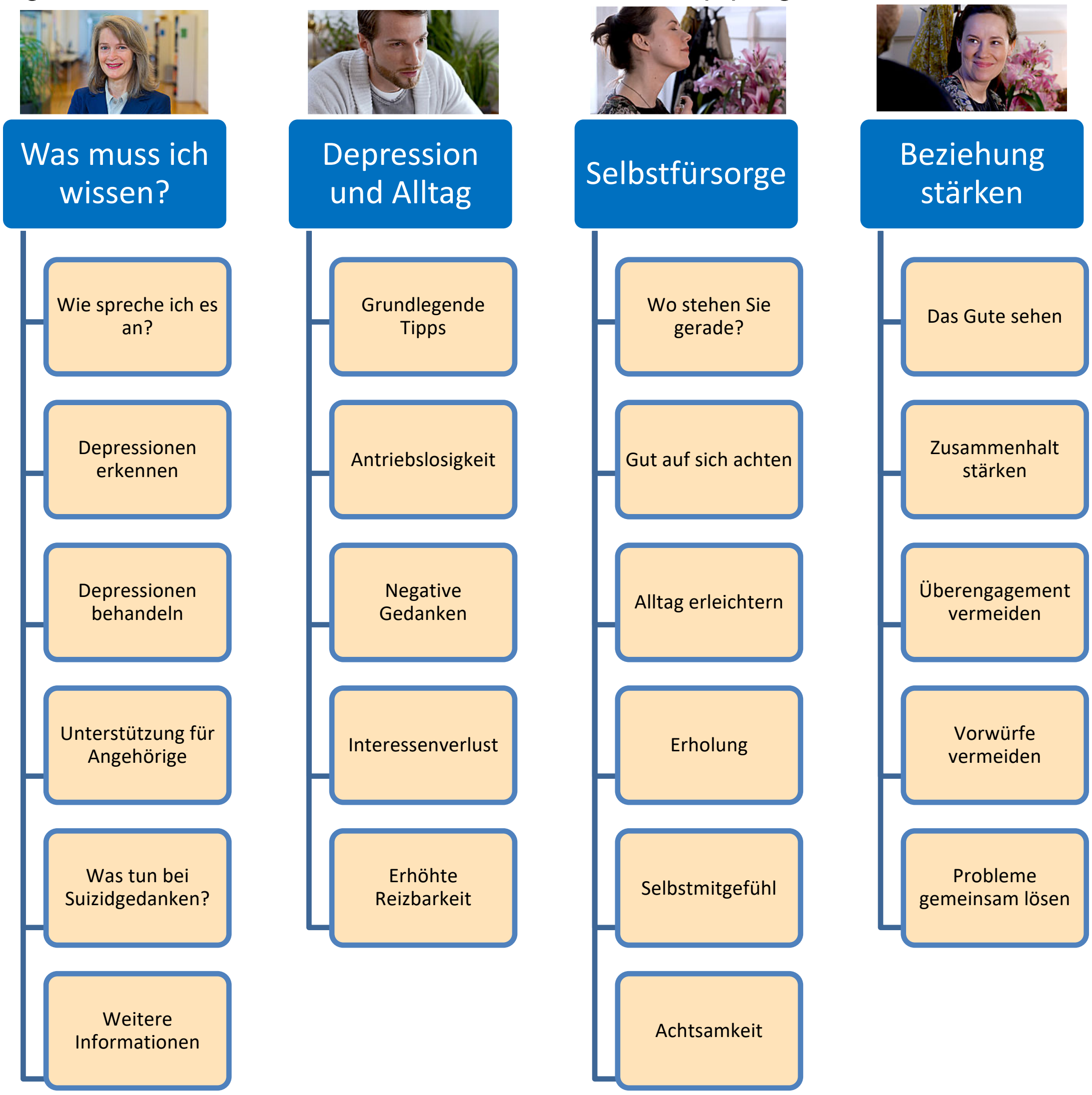

During the study period of four weeks, the online self-help program will be offered in the following three individually randomized variants:

1) *Online self-help program with individual support*: The individual e-mail-support will be implemented by specifically trained psychologists and under regular supervision

with experienced psychotherapists. The psychologist contacts the caregivers three time per week via a secure message system, to give additional information about the modules (e.g. using examples), clarify individual content-related questions and strengthen the motivation of the caregivers in dealing with individual obstacles. For the supporting psychologists, guidelines with instructions and text templates will be developed, to reduce the expenditures of time and costs substantially. Because of its high degree of standardisation, the email-support is much more efficient than other possible formats (e.g. telephone or chat). Since many questions will arise repetitively, text templates will be created gradually, which will reduce personnel costs for the individual support.

2) *Online self-help program with automated support:* The fully automated message system includes regular motivational e-mails, reminders with encouraging messages, and feedback on completed modules. In the development of the automated support system, a focus group consisting of experts in e-mental-health and psychoeducation for relatives was conducted, and caregiver experiences were collected in qualitative interviews in a pilot study.

3) *treatment as usual (TAU) control condition with written information*: In the TAU control condition the caregivers receive a digital version of the patient information leaflet „Depression – Angehörige und Freunde“, which was developed by the ÄZQ based on patient guidelines of unipolar depression.(Ärztliches Zentrum für Qualität in der Medizin (ÄZQ), 2016). On two pages, this document presents concise information on living with the situation, supporting the depressed person, managing crises and caring for oneself. After the study period of three months, the caregivers of the TAU control condition can also use the online self-help program with automated support.

4.4 Outcomes

Primary outcome:

The primary outcome of the study is the unspecific psychological distress as indicator for the risk of mental illness in caregivers (K-10-scale) at post-intervention (T2).

Secondary outcomes:

All outcomes are to be assessed at post-intervention (T2) and at 3-month follow-up (T3)

- The subjective und objective psychosocial burden of caregiving (involvement evaluation questionnaire / IEQ-EU)
- The subjective symptom burden (symptom checklist short version / SCL-K-9)
- The depression literacy (depression literacy test; D-Lit)
- High expressed emotion aspects “critical comments” and “over-involvement” (Familienfragebogen; FFB)
- The well-being (WHO-5) of the caregivers
- Depressive symptoms in the depressed person (patient health questionnaire PHQ-9).

4.5 Participant timeline

Before registration on the website, interested caregivers are provided with written information on the study and an e-mail-address of the study team to answer any remaining questions. Informed consent is obtained from participants by actively selecting a checkbox at the end of the informed consent and data protection summary. Afterwards, caregivers can register on the website by filling in their e-mail-address and responding to a verification e-mail (double opt in). Afterwards, the caregiver will be asked some questions to check the in- and exclusion criteria (Screening). If the caregiver fulfills the criteria, he/she will be included in the study and asked, if the depressed person would like to participate as well. At the caregiver’s request, a personalized invitation e-mail is sent to the depressed person, which directs him/her to a study information and informed consent page tailored at depressed participants. Then the caregiver (and if applicable the depressed person) will be asked to complete the pre-intervention questionnaires (T0). After completing his/her questionnaires, the caregiver will be randomized (IND:AUT:TAU; 2:2:1) to one of the three interventions:

1) **Online self-help program with individual support (IND):**

The caregiver can now use the online self-help program for four weeks. He/she receives additional messages from psychologist three times per week, to give additional information about the modules (e.g. using examples), clarify individual content-related questions and strength the motivation of the caregivers in dealing with individual obstacles. The caregiver can contact the psychologist by writing messages in the same secure system.

2) **Online self-help program with automated support (AUT):**

The caregiver can now use the online self-help program for four weeks. He/she receives additional fully automated messages included regular motivational e-mails, reminders with encouraging messages, and feedback on completed modules. The caregiver cannot reply to the automated messages sent by the online program.

3) **treatment as usual control condition with written information (TAU):**

The caregiver gets access to the patient information „Depression – Angehörige und Freunde“, which is developed by the ÄZQ based on patient guidelines of unipolar depression. After the study period of three month the caregivers of the TAU-control-condition can also use the online self-help program with automated support.

One week after randomization (T1) the caregiver will be asked to fill in a short questionnaire concerning the psychological distress of the caregivers (K-10). After the intervention period of four weeks, the caregivers and depressed persons fill in the psychological questionnaires again (T2). At this time, there will be additional questionnaire for the caregivers who were randomized to one of the active interventions, asking for the acceptance, adherence to and usage of the online program. All caregivers are also asked, if adverse events occurred during the intervention period. Three months after T0 (T3) the caregivers and depressed persons will fill in the psychological questionnaires again und the caregivers will be asked for occurred adverse events in the last 8 weeks.

At T2 and optionally T3, individual participants (caregivers and depressed persons) will be selected based on stratification criteria and invited to take part in a research interview of approximately 60-90 minutes by telephone. The participation in the interview is voluntary and does not affect the general study participation in any way. If the participants are interested in taking part, they will be given an additional study information and consent form for the interview.

The participant's timeline is shown in the following figure:

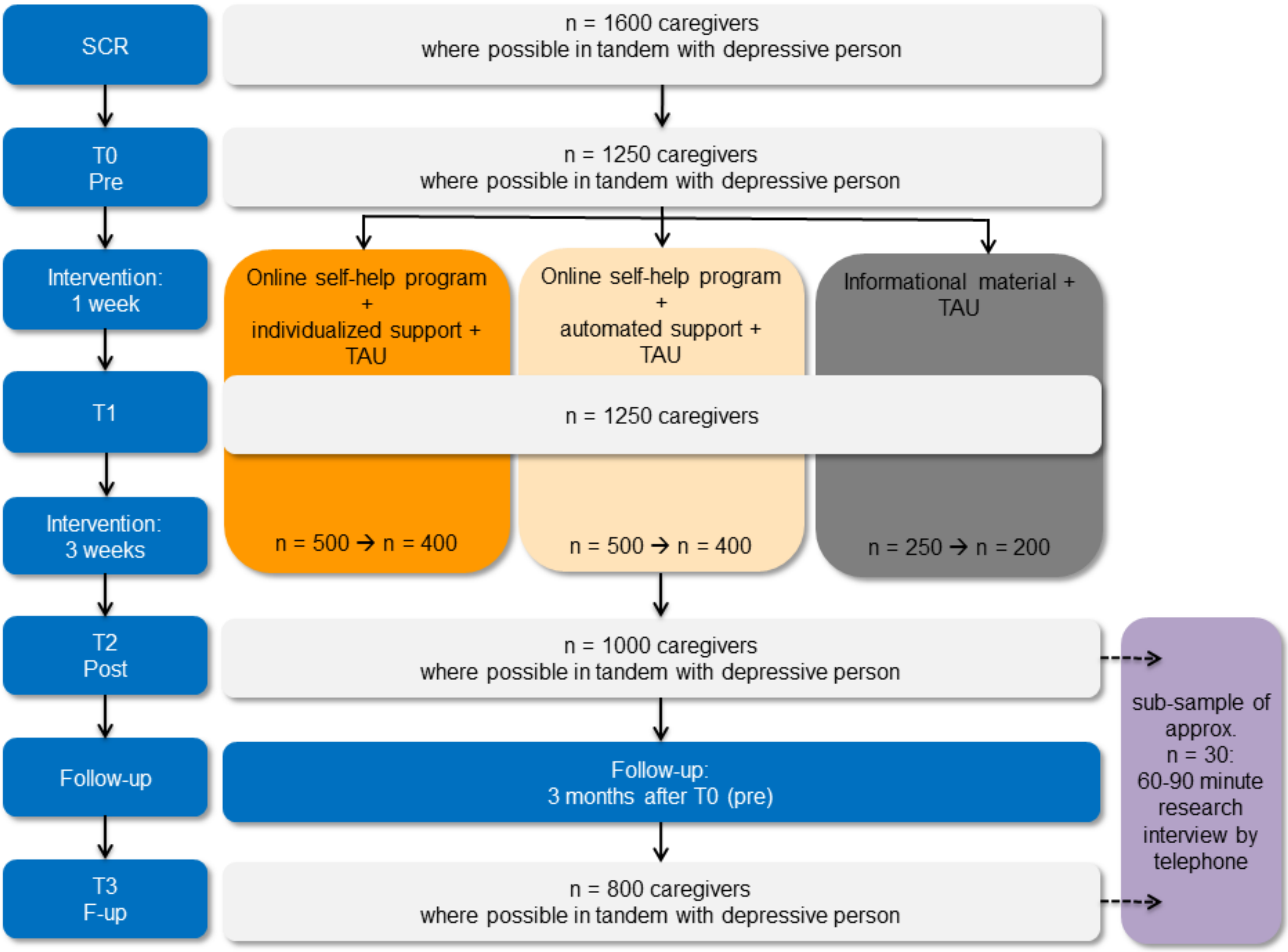

4.6 Sample Size

The numbers of caregivers to be randomized to the respective intervention and control groups IND:AUT:TAU are n=500:500:250. This accounts for an expected 20% rate of missing K-10 outcome measurements at T2 – yielding an expected n=400:400:200 non-missing primary outcomes – and for the following power considerations. Randomized groups will be compared using a sequential closed test procedure: To guarantee a multiple significance level of 5%, confirmatory statistical testing at nominal significance level α=5% will continue until the first non-significant result. Pairwise comparisons between randomized groups will be performed via calculation of a two-sided 95% confidence interval for the difference of means, for which exclusion of a zero difference is equivalent to rejection of the null hypothesis in an unpaired t-test of equal means at two-sided significance level α=5%. The first and second statistical test to reject equalities IND=TAU and AUT=TAU of mean pre-to-

post-(T2-T0) K-10 changes at significance level α=5% will implement the primary objective by comparing online self-help program interventions with control; they have a power of 1-β≈99% given a medium effect size (Cohen's d) of d=0.5, and of 1-β=93% if d=0.3. Such effect sizes appear to be realistic based on a single-arm pre-post comparison following a psychoeducation program which yielded d=1.1 for a shorter version of the primary outcome scale, the K-6 scale (Katsuki et al., 2011). As regards the third comparison among the two online self-help program intervention groups IND and AUT, both superiority of IND over AUT and, to a lesser degree, equality of IND and AUT can appear realistic; it would be worth-while to reject equality IND=AUT or inferiority of AUT. The pairwise test comparing the two online self-help program intervention groups to reject IND=AUT has a power of 1-β=81%, given a small effect size of d=0.2. Confirmatory testing will stop after this comparison. Given equal means IND=AUT (d=0), it would also be of interest to reject inferiority of AUT (d=-0.1), i.e. to demonstrate non-inferiority of the less costly intervention AUT compared to the more costly intervention IND at one-sided significance level α=2.5%. The power to achieve this secondary objective, i.e. to reject such a small inferiority, is 1-β=29%. Higher recruitment targets to achieve greater power were dismissed for reasons of feasibility. The scenarios considered are summarized in terms of standardized group means shifted towards 0 for the TAU group in the table below (nQuery V8.3.1.0, module MGT0U).

Standardised mean IND,AUT,TAU	Power to reject a single null hypothesis (n=400:400:200):			
	IND=TAU	AUT=TAU	IND=AUT	AUT inf IND
0.5, 0.3, 0	99%	93%	81%	<1%
0.5, 0.5, 0	99%	99%	5%	29%
0.3, 0.3, 0	93%	93%	5%	29%

Notes: inf refers to the null hypothesis that AUT is inferior to IND (d=-0.1).

4.7 Recruitment

Caregivers of depressed persons will be recruited online throughout Germany. As the majority of depressed persons are treated by general practitioners (Gerste & Roick, 2014), general practitioner practices as well as specialist practices for psychiatry and psychotherapy and psychotherapists will be informed and sensitized to the role of caregivers in the treatment of depression. Clinics for psychiatry and psychotherapy will be included in the recruitment strategy as well, because within in-patient care only the

minority of caregivers is able to access psychoeducative offers, so most caregivers are unprepared for the critical phase after the discharge of the depressed patient. We will provide informational material for patients and care-givers (posters, flyers) to interested practices and clinics. Other caregivers will be addressed through online forums, self-help groups for caregivers, self-help contact bureaus, crisis intervention centers and caregivers' associations. Furthermore, information on study participation will be disseminated through 85 regional alliances against depression (Bündnisse gegen Depression) and a patient congress (most recently in Leipzig with more than 1300 participants, half of which caregivers of depressed persons), both organized by the German Depression Assistance Foundation (Stiftung Deutsche Depressionshilfe), as well as through a dedicated informational web page for relatives of depressed persons by the German Depression Assistance Foundation which has several hundred visitors each day. The general public will be addressed through the members' magazines and internet pages of the cooperating health insurance AOK and via advertisements in search engines, social platforms and other media.

The informed consent, delivery of the intervention and data collection will take place on a research platform website set up for these purposes. No information on participants will be processed or communicated during recruitment. Rather, potential participants will be referred to the research website for information and registration.

It is expected that during the twenty-two-month recruiting period about 1600 caregivers will be declared about the study and screened in terms of the inclusion criteria and that 1250 caregivers can be included and randomized within the study.

5 Methods: Assignment of interventions

5.1 Allocation

5.1.1 Sequence generation

For allocation to the intervention and control groups IND, AUT and TAU, a computer-generated random list with a 2:2:1 ratio is used, with stratification based on the participating caregivers characteristics as follows:

- age (years, 18-40, 41-65, ≥ 66)
- gender (female, male, diverse)
- relation with depressed person (parent, child, partner, other)
- K-10-scale (10-22, 23-50).

These characteristics were selected because each variable could plausibly influence participants' response to the interventions. The K-10 cut-off value was chosen based on its ability to differentiate healthy persons from persons at risk of a mental disorder in the German validation study (Giesinger, Rumpold, & Schüßler, 2008). Details of the randomization algorithm will be documented separately and will not be disclosed to the participants.

5.1.2 Allocation concealment mechanism

The allocation sequence is implemented in the designated online platform. The result of randomization is communicated to the caregivers via internet after informed consent, screening, and entry of their baseline data. Randomization lists are only accessible to the technical administrators of the online platform.

5.1.3 Implementation

Randomization lists per stratum are generated by IMBI staff not involved in the trial using R version 4.0.2 or higher and supplied to the technical administrators of the online platform. Participating caregivers enroll themselves into the trial during the online informed consent process and are automatically allocated online to one of the three arms using the caregiver's baseline data for stratification.

5.2 Blinding (masking)

This is an open-label trial, therefore, there is no blinding, neither for participants nor for the study team.

6 Methods: Date collection, management and analysis

6.1 Data collection methods

6.1.1 General Data

By registration on the website the caregiver needs to fill in his/her e-mail-address and user name. Afterwards the caregiver will be asked some questions to check the in- and exclusion criteria (Screening). If the caregiver fulfils the criteria, he/she will be included in the study and asked, if the depressed person would like to participate as well. Then the caregiver will be asked for some general data including sociodemographic data of the caregiver and the depressed person, internet literacy, prior experience with psychoeducation as well as diagnostic and treatment status of the depressed person.

Before the intervention period starts the participant has to fill in the psychological questionnaires at T0 (see 0). If the depressed person participates as well, he/she will be asked to fill in a short questionnaire concerning his/her depressive symptoms. One week after starting the intervention (T1) the caregivers will be asked to fill in a short questionnaire concerning the psychological distress of the caregivers (K-10). After the intervention period of four weeks the caregivers and depressed persons fill in the psychological questionnaires again (T2). At this time there will be an additional questionnaire for caregivers asking for the acceptance, adherence and usage of the online program. All caregivers were also asked, if adverse events were occurring during the intervention period. Three months after T0 (T3) the caregivers and depressed persons will fill in the psychological questionnaires again und the caregivers will be asked for occurred adverse events in the last 8 weeks.

6.1.2 Psychological variables

The psychological variables will be collected by the following questionnaires (or interview), which the caregivers fill in (or participate in) at T0, T1 (only K-10), T2 and T3:

	Screening	T0	T1	T2	T3
**Caregiver**					
informed consent	x				
Screening – questions concerning in- and exclusion criteria	x				
sociodemographic data and relationship with the depressed person		x			
internet literacy		x			
K-10		x	x	x	x
IEQ-EU		x		x	x
SCL-K-9		x		x	x
D-Lit		x		x	x
FFB		x		x	x
WHO-5		x		x	x
acceptance, adherence and usages questionnaire				x	
adverse events questionnaire				x	x
Qualitative interview				(x)	(x)
**depressed person (if applicable)**					
informed consent	x				
PHQ-9		x		x	x
Qualitative interview				(x)	(x)

K10 Scale: The K10 scale (Kessler et al., 2002; German version: Giesinger, Rumpold, & Schüßler, 2008) assesses non-specific psychological distress during the past 30 days. It includes ten items on a five-point Likert scale and five additional items. The answer options for the first ten items range from one (none of the time) to five (all of the time). Questions begin with "During the last 30 days, about how often did …" and continue with (1) … you feel depressed (2) … you feel tired out for no good reason? (3) … you feel nervous?" The maximum score is 50, indicating high psychological

distress. Ten, the minimum score, indicates the absence of psychological distress. The additional items relate to the frequency of psychological distress in the past 30 days as compared to other times, the performance, the frequency of medical visits and the attribution of physical diseases on the perceived distress.

Giesinger et al. (2008) report good internal consistency of the scale (Cronbachs Alpha ranging from 0.80 – 0.90). To measure convergent validity, satisfactory correlations with related scales could be found (correlation with the State-Anxiety-Scale of the STAI (Laux, L., Glanzmann, P., Schaffner, P., Spielberger, 1981) r = .68 and with the scale GSI of the BSI (Franke, 2000) r = .71)

The Involvement Evaluation Questionnaire: **The Involvement Evaluation Questionnaire**: The IEQ-EU (van Wijngaarden et al., 2000; van Winjgaarden, 1992) assessed the burden on caregivers of mentally ill people in the past four weeks. The present study used the German version of the IEQ-EU (Bernert et al., 2001). The IEQ-EU consists of 31 items, which are assessed on a five-point Likert scale ranging from "never" to "always". Of the 31 items, 27 are grouped into four subscales, namely tension, supervision, worrying, and urging. The internal consistency (Cronbachs Alpha) of the subscales is reported as moderate or good, ranging from 0.71- 0.88, for the German version (Bernert et al., 2001).

Symptom checklist 9-item short version: The psychological burden on caregivers of mentally ill people was assessed by the German version of the SCL-K-9 (Klaghofer, R., Brähler, 2001) a shortened version of the Symptom Checklist 90-R (Derogatis, 1977; Franke, 1992). By using nine items on a five-point Likert scale ranging from 0 (not at all) to 4 (very severe), different psychological symptoms are assessed. Cronbach's Alpha was α = 0.87, which stands for a good reliability of the SCL-K-9 (Petrowski, Schmalbach, Kliem, Hinz, & Brähler, 2019).

Depression literacy test: The literacy concerning depression was assessed, using the German version of the Depression Literacy Test (D-Lit) (Freitag et al., 2018; english original: Griffiths, K.M., Christensen, H., Jorm, A.F., Evans, K., Groves, 2004). The D-Lit is a 35-item self-report questionnaire, assessing general information about depressive disorders, for example symptoms, impairments and treatments. Response options are "true", "false" and "don't know". Reliability of the scale is reported as moderate with a Cronbachs Alpha of 0.75 (Freitag et al., 2018).

Family Questionnaire: The level of expressed emotion (EE) was assessed by the German version of the Family Questionnaire (FQ) (Wiedemann, Rayki, Feinstein, &

Hahlweg, 2002) a 20-item self-report questionnaire. On a four-point likert scale, ranging from “never” to “very often” caregivers of patients evaluate the frequency of specific reactions toward the patient. Internal consistency was excellent for the subscale “criticism” (Cronbachs Alpha ranging from 0.90- 0.92 depending on the sample) and good for the subscale “emotional over-involvement” (Cronbachs Alpha ranging from 0.79-0.82 depending on the sample).

WHO-5: The subjective psychological well-being was assessed by the German version of the 5-item World Health Organization Well-Being Index (WHO-5) (WHO, 1998). Response options are coded on a six point scale (0 = never; 5 = all of the time). The internal consistency of the German version of the WHO-5 is excellent (Cronbachs Alpha = 0.92) (Brähler, Mühlan, Albani, & Schmidt, 2007).

If the depressed person participates in the study as well, the person has to reply the following questionnaire at T0, T2 and T3:

Patient health questionnaire: Depressive symptoms were assessed by the German version of the patient health questionnaire (PHQ-9) (Löwe, B., Spitzer, R.L., Zipfel, S., Herzog, 2002; english original: Spitzer, 1999). Subjects indicated for each of the nine items on a four point scale, ranging from “never” to “nearly every day”, whether the symptom has bothered them during the previous 2 weeks.

The PHQ-9 showed excellent criterion validity (medical patients: sensitivity, 95%; specificity, 86%) an good internal consistency (Cronbachs Alpha = 0.88) (Gräfe, Zipfel, Herzog, & Löwe, 2004).

6.1.3 Usage Data of the online program

The following usage data will be collected by the online program for each participant: individual progress in the four modules, dates and frequencies of logins and time spent in the online program. In the condition with individualized support, the correspondence with the psychologist will be stored as well, and after manually removing any potentially identifying information, will be used for content analyses of topics in support messages in pseudonymized form.

6.1.4 Data collection quality

To improve data completeness the participants will get repeated E-Mail reminders, if they don´t fill in the questionnaires at T1, T2 or T3. For completing all measurements, the caregivers get a compensation in the form of a 30€ voucher (10€ for each measurement point, to be traded in at a large number of services).

If participants choose to end participation in the study, they will be asked to provide their reasons. Participants no longer interested in the intervention will nonetheless be asked to complete all measurements.

To promote data quality, data will be monitored during data collection. Data monitoring will consist of blinded reviews of the data for programming errors (e. g., data ranges different than expected) as well as for missing data. If problems are detected, corresponding steps will be taken (e. g., additional efforts to reach participants for each measurement if a large proportion of data is found to be missing).

6.1.5 Collection of qualitative interview data

At T2 and possibly T3, individual participants (caregivers and depressed persons) will be selected based on stratification criteria (based on theoretical sampling with both a priori criteria such as type of relationship to depressed person as well as criteria emerging in the analysis process) and interviewed by telephone for approximately 60-90 minutes. The interviews are conducted by trained psychological personnel using a semi-structured interview guideline. The contents of the interviews with caregivers focus on the experiences with the online self-help program, the support conditions, everyday life experiences and the interaction with the depressed person as well as potential changes brought about by the intervention. The contents of the interviews with depressed persons focus on the depressed persons' experiences of potential changes in the caregivers' behavior and their interactions. The semi-structured interview form is intended to capture the subjective perception of the participants. It is characterised by a predetermined catalogue of questions with the flexibility to elaborate topics that arise during the interview or change the order of questions to follow the flow of the conversation. The interview consists of a warm-up phase and five thematic blocks. After the general experiences with the coach and the supportive messages, specific experiences with the topic areas of all four modules will be asked. There are a few narrative prompts for each thematic block, supplemented by follow-up questions that are only explicitly asked if the respective aspects do not appear in the participants' spontaneous narrative or report. The content of the interviews is recorded in the form of audio recordings. Before further analysis, the audio recordings are transcribed into text, removing or changing names and other potentially identifying information to achieve pseudonymized data.

6.2 Data management

6.2.1 Storage of data

All data collected in the context of this study will be collected electronically only. Descriptive data of the participants and usage data (e.g. login duration and frequency) will be recorded, as well as data from questionnaire surveys. The data collection takes place on the website of the online self-help-program, which is created and operated (hosting, account management) by the consortium partner H6 Kommunikationsagentur (Geusenstraße 8, 10317 Berlin, Germany) under the leadership of Prof. Gabor Kovacs. For technical and administrative processes H6 Kommunikationsagentur has access to all data collected in the study. All data collected in the study will be stored on a server of the company Mittwald CM Service GmbH & Co. KG (Königsberger Straße 4-6, 32339 Espelkamp, Germany).

Compliance with the European General Data Protection Regulation (GDPR: EU Regulation 2016/679) is secured through a data processor agreement (AV-Vertrag) between Mittwald and the University Clinic. The same applies for other potential contractors of the consortium partners, while shared and individual responsibilities between the consortium partners H6 Kommunikationsagentur and the University Clinic will be defined in a contract of shared responsibilities (according to Art. 26 DS-GVO).

Data files will be archived in order to comply with the data protection requirements and deleted completely after 10 years. The required archiving periods of at least five years after publications will be adhered to.

The correspondence between the particpants in the condition with individualized support and the psychologist will be transferred by encrypted connection to secure clinic servers for storage and further processing. Before the evaluation of this data, the correspondence are checked by study staff to see whether information appears that allows conclusions to be drawn about the caregiver or the depressed person (e.g. names, addresses, ...). If this is the case, the information is deleted from the correspondence.

The contents of the interviews are divided into sensitive audio recordings on the one hand, which remain on protected clinic servers, and pseudonymized transcripts (names and other information that make a person identifiable are changed during transcription) on the other hand for the further research process. Strict separation of the subjects' names or audio recordings and the other data collected ensures pseudonymized data processing. In case that subjects request a complete deletion of

their own data, all personal data will be deleted. Accordingly, deletion includes all demographic data, contact data and data of inclusion and exclusion criteria as well as potential interview recordings. Questionnaire data, pseudonymized psychological correspondence and transcribed interview data, that has been produced until then, will be analyzed completely anonymized.

6.2.2 Data Transfer

The stored data will be compiled for analysis by the consortium partner H6 Kommunikationsagentur and transferred only to the Institute of Medical Biometry and Statistics of the University Medical Center Freiburg (IMBI) and the study direction. Data transfer to other parties is not intended.

In order to analyse the data, the data records will be transmitted to the Institute of Medical Biometry and Statistics in pseudonymized form. During data collection the Institute of Medical Biometry and Statistics gets access to the data, to monitor the data quality. For further monitoring, the principal investigator can request a data transfer of the pseudonymized data at any time. Data transfer will be carried out only electronically via encrypted data connections and only on the instructions of the principal investigator.

6.2.3 Data protection/Data security

To participate in the study, the participants register with a confirmed e-mail address and a secure password. The use of the online program, messaging system and the associated data collection will take place exclusively in the protected login area of the online program. All data collected will be stored by the Mittwald company. The Mittwald Company undertakes to perpetually treat the collected data confidentially and to use it only for the purpose of conducting the present study. Personal data will be processed in such a way that the data can no longer be assigned to a specific subject without additional information (pseudonymization).

The company Mittwald gives a written undertaking to comply with the European General Data Protection Regulation (GDPR: EU Regulation 2016/679) and with the technical and organizational measures being necessary and appropriate for their compliance, which are as follows:

No unauthorized access to technical facilities	– The company building is divided into different areas of access.<br>– Visitors must register at the reception and are picked up by their contact person.<br>– Access to all data processing systems will be completely denied to unauthorized persons.<br>– The access of any person (including employees) must be approved in advance by authorized personnel and is verified by checks on persons.<br>– All accesses and premises of the data processing systems will be monitored by cameras and controlled by electronic locking systems.<br>– All access will be recorded.
Access controls to important facilities (hardware, operating systems, software)	– The access to the data processing systems is secured by a user and rights administration. Employees can only view, use, process or delete the data required for their tasks.<br>– Accesses to the data processing systems will be logged.<br>– When leaving the workplace, the system is blocked by a screensaver and can be unlocked only by entering the password.<br>– Accordingly, at the time of recruitment every employee is committed to confidentiality and compliance with data protection. A violation would result in instant dismissal and a criminal complaint.
Control of data transmission	– Personal data will be transmitted only electronically via encrypted data connections, so that they cannot be read, copied, changed, or removed by unauthorized persons.

	- Data media that are no longer required or defective will be disposed of by a certified company.
Data Transfer	All Data transfer will be encrypted (encrypted Internet connections using TLS/SSL).

If, despite the detailed technical and organizational measures taken, there should be a serious disturbance of operating procedures, a suspicion of data protection violations or other irregularities in the processing of personal data, the consortiums partners are obliged to inform the study direction immediately and to jointly decide on further proceedings.

All data processing will be carried out in Germany or a member state of the European Union only.

Mittwald Company also implements a data protection management system for the regular monitoring, assessment and evaluation of data protection and the effectiveness of the established technical and organizational measures.

6.2.4 Data integrity

To ensure data integrity, it can be checked retrospectively at any time whether and by whom personal data has been entered, changed or removed in data processing systems. The entry, modification or deletion of personal data is logged with the identification of the responsible employee.

Additionally, the protection of personal data against accidental destruction or loss is ensured (e.g. by RAID systems, replacement hardware, surge protection, UPS systems, emergency generator, extinguishing systems). Also, measures will be taken to ensure the quick restoration of availability and access to personal data in the case of a physical or technical incident (e.g. by daily backups).

6.3 Statistical methods

6.3.1 Biostatistical planning and analysis

Before the start of the final analysis, the details of statistical analysis left open in the trial protocol will be described in a statistical analysis plan (SAP). At the latest, the SAP will be completed during a blinded review of the trial data, i.e. without looking at the randomized intervention for individual caregivers. If the SAP contains any changes to the analyses outlined in the trial protocol, they will be marked as such, and reasons for amendments will be given.

Throughout, emphasis will be given to reporting of effect estimators with two-sided 95%-confidence intervals rather than p-values whenever possible, which will be considered statistically significant if below 5% for tests of equality. However, confirmatory statistical testing will be restricted to the analysis of the primary outcome, to be followed by descriptive reporting of other trial results.

All programming for statistical analysis will be performed with the Statistical Analysis System (SAS) or with R.

6.3.2 Main analysis of primary outcome

Model: The primary efficacy analysis will be performed in the full analysis set (FAS) according to the intention-to-treat (ITT) principle. Therefore, all randomized caregivers will be analysed in the assigned treatment arms, irrespective of treatment adherence and whether they refuse or discontinue the intervention. The effects of allocation to IND, AUT and TAU with respect to the primary endpoint, change in K-10 score from baseline to four weeks after randomisation, will be estimated and tested in a linear regression model, and the corresponding two-sided 95%-confidence intervals will be reported. The model will include randomized treatment (IND, AUT, TAU), age (years, 18-40, 41-65, ≥ 66), gender (female, male; for diverse, see end of paragraph), caregiver's relation with depressed person (parent, child, partner, other), and K-10 baseline scores as independent variables. Since very few caregivers of diverse gender are expected, they will be assigned alternatingly to the groups of females and males in the primary analysis of intervention effects. They will be analysed separately in secondary analyses exploring the effect of gender.

Sequential testing: Following a sequential closed testing procedure to ensure a multiple type I error rate of 5%, confirmatory comparisons of randomized treatment arms will proceed until the first occurrence of a non-significant result at the nominal 5% level, followed by descriptive reporting of all subsequent analyses. The two-sided tests of equality of means of two treatment arms will be based on the two-sided 95% confidence interval for the difference in mean change from baseline estimated from the linear regression model. Treatment arms will be compared in the following order: IND versus TAU, AUT versus TAU, IND versus AUT. Although it is hypothesized and subjected to confirmatory testing that IND is superior to AUT, equality is also deemed possible. In a secondary descriptive analysis, demonstration of non-inferiority of AUT compared to IND will also be attempted. If the lower limit of the two-sided 95%-confidence interval for the difference of mean K-10 changes from baseline (IND minus

AUT) is greater than -0.62 points, this will be interpreted as non-inferiority. The non-inferiority margin was derived as follows: For caregivers of depressed persons, the expected standard deviation of K-10 scores is in between that of patients (SD: 4.6 points) and students (SD: 7.8 points) seen in the German norm sample (Giesinger et al., 2008). Therefore, a standard deviation of 6.2 score points is expected. Assuming a correlation of 0.5 between baseline and post-intervention K-10 scores, the change from baseline has also a standard deviation of 6.2 points, and using Cohen's d=0.1 as non-inferiority margin gives 0.62 points.

Handling of missing values: Multiple imputation will be employed to replace missing values. Imputation will be done per randomized treatment arm, using baseline data and post-baseline information according to a Treatment Policy Strategy as described by Polverejan and Dragalin (2020) and Guizzaro et al. (2020). The results will be combined using Rubin's rules for multiple imputation. The details will be fixed in the statistical analysis plan.

Assumptions: The resulting estimators assume that caregivers with missing outcome data have outcomes similar to those of subjects with similar baseline data and treatment adherence who provide their outcome data.

Interim analysis: As described in section 7.1, an interim analysis is not considered necessary for this study. The lead investigator will monitor the progress of the study with regard to safety-relevant development as detailed in section 7.2.

6.3.3 Sensitivity analysis of primary outcome

To evaluate the robustness of the main analysis with respect to the assumptions on missingness, an alternative multiple imputation model will be applied for sensitivity analysis. It will impute the outcomes that are missing due to study withdrawal such that outcomes in the IND and AUT arms are assumed to be similar to those observed in the TAU group. The details will be fixed in the statistical analysis plan.

To evaluate the robustness of the comparison of IND versus AUT in the main analysis with respect to adherence, the main analysis will be repeated in subpopulations of adherent caregivers. Non-inferiority will be concluded if the lower limit of the corresponding two-sided 95%-confidence interval for the difference of mean K-10 changes from baseline (IND minus AUT) is greater than -0.62 points. The details will be fixed in the statistical analysis plan.

6.3.4 Supplementary analysis of primary outcome

In order to assess the consistency of the treatment effect in relevant subgroups (EMA-CHMP, 2019), exploratory analyses will be performed using all variables for grouping that were used for stratified randomization (i. e. age, gender, relation with depressed person, pre-intervention (T0) K-10 score) as well as internet literacy.

Further analyses will explore possible moderators (measured at baseline, e. g., possible effects of features of the depressed person such as duration of illness or type of relation with the depressed person) and mediators (e. g. adherence to randomized interventions, importance of changes in interaction behaviour for treatment effects on participants K-10 scores) on primary and secondary outcomes. These analyses will be conducted as part of PhD theses and will be pre-specified elsewhere at a later time.

6.3.5 Analysis of secondary outcomes

Secondary outcomes at T2, four weeks after randomization, and T3, three months after randomization, will be evaluated in a linear mixed model per outcome scale, with a compound symmetry covariance matrix to account for correlation between T2- and T3-outcomes of the same caregiver or depressed person. Independent variables will be those of the main primary analysis, plus time point (T2, T3). The details will be fixed in the statistical analysis plan.

Subgroup analyses, moderator and mediator analyses will be performed as described for the primary outcome above.

Furthermore, the results seen in the Facing Depression Together trial will be compared to historical controls of face-to-face psychoeducation groups for caregivers of depressive in-patients from the multicentre SCHILD study (Frank, Wilk, et al., 2015).

6.3.6 Further supplementary analyses

Demographic and other baseline data will be summarised descriptively by randomized arm in the FAS. Continuous data will be summarised by arithmetic mean, standard deviation, minimum, 25% quantile, median, 75% quantile, maximum, and the number of complete and missing observations. If appropriate, continuous variables can also be presented in categories. Categorical data will be summarised by the total number of patients in each category and the number of missing values. Caregivers frequencies are displayed as valid % (number of patients divided by the number of patients with non-missing values).

All data items needed for reporting of trial results in compliance with the CONSORT (Schulz, Altman, & Moher, 2010) and CONSORT-EHEALTH (Eysenbach & CONSORT-EHEALTH Group, 2011) statements will be evaluated.

6.4 Qualitative methods

Based in the interview transcripts, a qualitative content analysis is carried out. The focus of the qualitative analysis is on the subjective perception of the interviewed person. Depending on the research question, an appropriate method within the grounded theory framework is selected. Our research group is experienced in both constructivist grounded theory analyses (Charmaz, 1996, 2006) and core sentence ("Kernsatz") analyses (Burkhardt, 2016; Leithäuser & Volmerg, 1988). For the messages between participants and psychologist from the individual support condition, an appropriate form of qualitative or quantitative content analysis is chosen for each research question.

7 Methods: Monitoring

7.1 Data Monitoring

Following the guideline on data monitoring committees (European Medicines Agency [EMA]) Committee for Medicinal Products for Human Use [CHMP], 2005), neither a data monitoring committee nor interim analyses are considered necessary for this study, given the non-patient participants, short intervention as well as study time and low risk of harm expected from the intervention. The lead investigator will monitor the progress of the study with regard to the safety-relevant development as detailed in section 7.2.

7.2 Harms

The objective of this online-coach is to effect a positive change of caregivers' psychological burden and everyday life. Each change includes a risk for undesirable side effects. Side effects can be impacts on everyday life, interpersonal relationships (e.g. conflicts due to caregivers setting more boundaries towards the patients) and increased awareness of the burdens. Moreover, a temporary burden through the active analysis of actual stress factors is possible. For risk assessment (serious) adverse events ((S)AE) will be defined ahead of the study and recorded during the course of

studies. (Serious) adverse events are the occurrence or deterioration of psychiatric symptoms of the caregiver or the depressive person.

In case of a (S)AE there is an emergency button on the website of the online program, where the participant can find information and contacts of emergencies services and will be asked to contact them.

If (S)AE occur in the psychological message system contact or if participants contact a study member in case of an (S)AE, the lead investigator will be informed of the (S)AE immediately and will decide on the further procedure (e.g. study exclusion and outpatient or inpatient treatment recommendations).

For the following reasons, the intervention will be ceased in subjects of the study:

- Revocation of the subject's declaration of consent
- If, in the opinion of the lead investigator, further participation in the study would have harmful effects on the subject or the depressive person
- In case of new exclusion criteria
- If the emotional burden for the subject is too serious to cope with it himself, and intensive (professional) support is needed

Exclusion is decided by the lead investigators, in case of fulfilling one of the mentioned criteria. The reason for exclusion will be recorded. If needed, excluded subjects will be supported in searching further consulting or treatment. Also, if the subject revokes the informed consent, the subsequent reason will be recorded.

The lead investigator will monitor the progress of the study with regard to safety-relevant development. The lead investigator will discontinue the study, if:

- the cost-benefit ratio for the caregiver or the depressive ill person changes significantly
- there are indications, that the security of the caregiver or the depressive ill person is no longer guaranteed
- the continuation of the study is no longer justified by ethical or medical reasons
- the questions of the study can be clearly answered by results of another study

7.3 Auditing

Since the trial is performed in non-patient participants (caregivers) in combination with a short intervention as well as study time and low risk of harm expected from the intervention, there will be no external audits.

8 Ethics and dissemination

8.1 Research ethics approval

Through working on the online self-help program and the messaging system the participating caregivers are expected to get better and more confident handling with the diagnosis depression and with the interpersonal interaction with the depressed person. Information and help is given, which applies in everyday life and the relationship. Also, the caregiver´s self-care is strengthened. Concerning the co-participating depressive person, it is expected, that trough the caregiver´s participation everyday interaction is getting more useful and less confrontational. Hence, it results a relief for the depressed person. As the online self-help program is a supportive and informing online-intervention, we expect that participation has a positive impact on everyday life and on the mental burden of the participating caregivers. In certain circumstances it may happen, that some subjects do just less or not benefit from the online-intervention. Since the results of this study help to better understand the burden of caregivers of depressive persons, all subjects help to improve possibilities for supporting caregivers of depressive persons.

The objective of this online self-help program is to effect a positive change of caregiver’s psychological burden and everyday life. Each change includes a risk for undesirable side effects. Side effects can be impacts on everyday life, interpersonal relationships (e.g. conflicts due to more delimitation) and experiencing of the burden. Moreover, a temporary burden through the active analysis of actual stress factors is possible. Subjects will be informed about all potential side effects by the informed consent.

During the whole study period, subjects can, if required (e.g. in case of a serious event), call a study member – or out of working hours – the doctor of the university clinic of psychiatry and psychotherapy, Freiburg.

Subjects will be informed about the study and all measures within the study. The participant consent (double opt-in) must be obtained before any trial-specific tests. Participation can be ceased at any time without necessarily giving any reason and without any loss of benefits in case of a (further) treatment at the university clinic Freiburg (independence of study and treatment).

The online self-help program is developed in accordance with the S3-guidlines of unipolar depression, which recommends psychoeducation for caregivers. The study is to be conducted according to all legal requirements, as well as ethic and medical

principals (Declaration of Helsinki, Good Clinical Practice, Data Protection Law). The Declaration of Helsinki is the standard for the medical and psychological ethic of the university clinic Freiburg.

8.2 Protocol amendments

Any modifications to the protocol which may impact on the conduct of the study, potential benefit of the participant or may affect participant safety, including changes of study objectives, study design, patient population, sample sizes, study procedures, or significant administrative aspects will require a formal amendment to the protocol. Such amendment will be approved by the Ethics Committee prior to implementation.

8.3 Consent or assent

After the registration on the website, the participant will be informed that participation is voluntary and that he/she may withdraw at any time without having to give reasons and without penalty or loss of benefits to which the participant is otherwise entitled.

The participant consent must be obtained before any trial-specific tests.

By agreeing the consent form, the participant agrees to voluntarily participate in the trial and declares that he/she agrees to the recording of personal data for the trial after pseudonymization.

Afterwards the participant will get the informed consent via E-Mail.

8.4 Confidentiality

The pertinent provisions on data protection must be fully complied with.

The study participants will be informed of the purpose and extent of the collection and use of personal data.

Findings obtained in the course of the trial will be stored on electronic media and treated in strict confidence. For the protection of these data, technical and organizational measures have been taken to prevent disclosure to unauthorized third parties. For example, the participant data will be captured in pseudonymized form (participant ID) throughout the documentation and evaluation phase. The participant IDs will be generated randomly, such that no information on the participant can be extracted from them (i. e., neither order of enrollment nor name initials etc. will be visible). Access to the identification list (connecting IDs and names etc.) is restricted to

the principal investigator. Where technical staff needs full access to the underlying databases, viewing of the identification list is precluded by organizational measures.

8.5 Declaration of interests

Prof. Schramm declares no potential conflicts of interest.

8.6 Access to data

The investigators grant access to participant files for verification of proper documentation of study data as required by law. The laws on data confidentiality (Art. 6 Abs. 1 lit. A DSGVO, LDSG Baden-Württemberg) apply in full. Persons authorized by the principal investigator are allowed to verify data.

No individual participant data is shared with the funding body or cooperating health insurance. The reporting duties towards the funding body are fulfilled with aggregated data (e. g. means, standard deviations) and statistical analyses.

8.7 Ancillary and post-trial care

As detailed in section 7.2, only mild and transient harms are plausibly expected from the planned interventions. Participants who encounter serious adverse events during the course of the study will be assisted in finding appropriate treatment where necessary.

8.8 Dissemination policy

Before recruitment and data collection starts, the trial will be registered at www.drks.de. Study results will be published in accordance to the criteria of the CONSORT-Statement. At least within one year of termination of the study, a manuscript for publication will be finalized. Any formal presentation or publication of data collected as a direct or indirect result of this trial will be considered as a joint publication by the investigators. It requires the agreement of the coordinating investigator and the co-principle investigator. Authorship will be determined by mutual agreement. The results of the study may be presented during scientific symposia or published in a scientific journal only after review and written approval by the coordinating investigator and the co-principle investigator. Investigators from the participating trial sites agree not to engage in presentations based on data gathered individually or by a subgroup of centers before publication of the first main publication, unless this has been agreed otherwise by all other investigators, the coordinating investigator and the co-principle investigator.

9 References

Andersson, G., & Titov, N. (2014). Advantages and limitations of Internet-based interventions for common mental disorders. *World Psychiatry*, *13*(1), 4–11. https://doi.org/10.1002/wps.20083

Ärztliches Zentrum für Qualität in der Medizin (ÄZQ). (2016). *Patienteninformation: Depression - Angehörige und Freunde [auf Basis der Patientenleitlinie „Unipolare Depression"]*. https://doi.org/10.6101/AZQ/000322

Bernert, S., Kilian, R., Matschinger, H., Mory, C., Roick, C., & Angermeyer, M. C. (2001). Die Erfassung der Belastung der Angehörigen psychisch erkrankter Menschen - Die deutsche Version des Involvement Evaluation Questionnaires (IEQ-EU) -. *Psychiatrische Praxis*, *28*(2), 97–101. https://doi.org/10.1055/s-2001-17792

Bischkopf, J., Wittmund, B., & Angermeyer, M. C. (2002). Alltag mit der Depression des Partners. *Psychotherapeut*, *47*(1), 11–15. https://doi.org/10.1007/s00278-001-0194-5

Brady, P., Kangas, M., & McGill, K. (2017). "Family Matters": A Systematic Review of the Evidence For Family Psychoeducation For Major Depressive Disorder. *Journal of Marital and Family Therapy*, *43*(2), 245–263. https://doi.org/10.1111/jmft.12204

Brähler, E., Mühlan, H., Albani, C., & Schmidt, S. (2007). Teststatistische Prüfung und Normierung der deutschen Versionen des EUROHIS-QOL Lebensqualität-Index und des WHO-5 Wohlbefindens-Index. *Diagnostica*, *53*(2), 83–96. https://doi.org/10.1026/0012-1924.53.2.83

Burkhardt, T. (2016). Identität und Migration. Auf der Suche nach Schutzfaktoren mit Problemzentrierten Interviews (PZI) und der Kernsatzmethode. In *Qualitative Methoden in der Sozialforschung* (pp. 145–151). Berlin, Heidelberg: Springer Berlin Heidelberg. https://doi.org/10.1007/978-3-662-47496-9_16

Butterworth, P., & Rodgers, B. (2006). Concordance in the mental health of spouses: analysis of a large national household panel survey. *Psychological Medicine*, *36*(05), 685. https://doi.org/10.1017/S0033291705006677

Charmaz, K. (1996). The Search for Meanings- Grounded Theory. In J. A. Smith, R. Harré, & L. van Langenhove (Eds.), *Rethinking Methods in Psychology* (pp. 27–49). London: Sage Publications.

Charmaz, K. (2006). *Constructing grounded theory*. Thousand Oaks: SAGE.
Derogatis, L. R. (1977). *SCL-90-R, administration, scoring & procedures manual-I for the R(evised) version*. Baltimore: John Hopkins University School of Medicine.
Deutsche Depressionshilfe. (2017). Deutschland-Barometer Depression. Volkskrankheit Depression – So denkt Deutschland. Retrieved from https://www.deutsche-depressionshilfe.de/presse-und-pr/downloads?file=files/cms/downloads/studienergebnisse_depression_so-denkt-deutschland.pdf
Deutsche Depressionshilfe. (2018). Befragung „Volkskrankheit Depression – So denkt Deutschland“. Retrieved December 18, 2018, from https://www.deutsche-depressionshilfe.de/presse-und-pr/downloads?file=files/cms/downloads/Barometer 2018/barometer-depression_grafikband-zum-download.pdf
DGPPN, BÄK, KBV, & AWMF (Hrsg.) für die Leitliniengruppe Unipolare Depression. (2015). *S3-Leitlinie / Nationale VersorgungsLeitlinie Unipolare Depression – Langfassung, 2. Auflage. Version 5. 2015*. https://doi.org/10.6101/AZQ/000364
Ebert, D. D., Van Daele, T., Nordgreen, T., Karekla, M., Compare, A., Zarbo, C., … Baumeister, H. (2018). Internet- and Mobile-Based Psychological Interventions: Applications, Efficacy, and Potential for Improving Mental Health. *European Psychologist*, *23*(2), 167–187. https://doi.org/10.1027/1016-9040/a000318
European Medicines Agency (EMA) Committee for Medicinal Products for Human Use (CHMP). (2005). Guideline on data monitoring committees (Doc. Ref. EMEA/CHMP/EWP/5872/03 Corr).
European Medicines Agency (EMA) Committee for Medicinal Products for Human Use (CHMP). (2019). Guideline on the investigation of subgroups in confirmatory clinical trials (Doc. Ref. EMA/CHMP/539146/2013).
Eysenbach, G., & CONSORT-EHEALTH Group. (2011). CONSORT-EHEALTH: Improving and standardizing evaluation reports of web-based and mobile health interventions. *Journal of Medical Internet Research*, *13*(4), e126. https://doi.org/10.2196/jmir.1923
Frank, F., Hasenmüller, M., Kaiser, M., Ries, Z., Bitzer, E., & Hölzel, L. (2015). Psychoedukative Gruppen für Angehörige von depressiv Erkrankten: Analyse des Informationsbedarfs durch eine Fokusgruppenuntersuchung. *PPmP - Psychotherapie · Psychosomatik · Medizinische Psychologie*, *65*(11), 426–433. https://doi.org/10.1055/s-0035-1555787
Frank, F., Rummel-Kluge, C., Berger, M., Bitzer, E. M., & Hölzel, L. P. (2014). Provision of group psychoeducation for relatives of persons in inpatient depression treatment - a cross-sectional survey of acute care hospitals in Germany. *BMC Psychiatry*, *14*(1), 1–9. https://doi.org/10.1186/1471-244X-14-143
Frank, F., Wilk, J., Kriston, L., Meister, R., Shimodera, S., Hesse, K., … Hölzel, L. P. (2015). Effectiveness of a brief psychoeducational group intervention for relatives on the course of disease in patients after inpatient depression treatment compared with treatment as usual - Study protocol of a multisite randomised controlled trial. *BMC Psychiatry*, *15*(1), 1–11. https://doi.org/10.1186/s12888-015-0633-4
Franke, G. H. (1992). Eine weitere Überprüfung der Symptom-Check-Liste (SCL-90-R) als Forschungsinstrument. *Diagnostica*, *38*, 160– 167.
Franke, G. H. (2000). *Brief Symtpom Inventory von L. R. Derogatis - Deutsche Version*. Göttingen: Beltz Test GmbH.
Franz, M., Meyer, T., & Gallhofer, B. (2003). Belastungen von Angehörigen schizophren und depressiv Erkrankter – eine repräsentative Erhebung. In J. M. Fegert & U. Ziegenhain (Eds.), *Hilfen für Alleinerziehende. Die Lebenssituation von Einelternfamilien in Deutschland* (pp. 215–229). Berlitz.
Freitag, S., Stolzenburg, S., Schomerus, G., & Schmidt, S. (2018). Depressionswissen – Deutsche Übersetzung und Testung der Depression Literacy Scale. *Psychiatrische Praxis*, *45*(08), 412–419. https://doi.org/10.1055/s-0043-119245
Gerste, B., & Roick, C. (2014). Prävalenz und Inzidenz sowie Versorgung depressiver Erkrankungen in Deutschland – Eine Analyse auf Basis der in Routinedaten dokumentierten Depressionsdiagnosen. In J. Klauber, C. Günster, B. Gerste, B.-P. Robra, & N. Schmacke (Eds.),

Versorgungs- Report 2013/2014 Schwerpunkt: Depression (pp. 21–54). Stuttgart: Schattauer.
Giesinger, J., Rumpold, M. G., & Schüßler, G. (2008). Die K10-Screening-Skala für unspezifischen psychischen Distress. *Psychosomatik Und Konsiliarpsychiatrie*, *2*(2), 104–111. https://doi.org/10.1007/s11800-008-0100-x
Gräfe, K., Zipfel, S., Herzog, W., & Löwe, B. (2004). Screening psychischer Störungen mit dem "Gesundheitsfragebogen für Patienten (PHQ-D)". *Diagnostica*, *50*(4), 171–181. https://doi.org/10.1026/0012-1924.50.4.171
Griffiths, K.M., Christensen, H., Jorm, A.F., Evans, K., Groves, C. (2004). Effect of web-based depression literacy and cognitive-behavioural therapy interventions on stigmatising attitudes to depression: Randomised controlled trial. *The British Journal of Psychiatry*, *185*, 342–349.
Guizzaro, L., Pétavy, F., Ristl, R., & Gallo, C. (2020). The use of a variable representing compliance improves accuracy of estimation of the effect of treatment allocation regardless of discontinuation in trials with incomplete follow-up. *Statistics in Biopharmaceutical Research*, 1–18. https://doi.org/10.1080/19466315.2020.1736141
Hölzel, L., Härter, M., Reese, C., & Kriston, L. (2011). Risk factors for chronic depression — A systematic review. *Journal of Affective Disorders*, *129*(1–3), 1–13. https://doi.org/10.1016/j.jad.2010.03.025
Idstad, M., Ask, H., & Tambs, K. (2010). Mental disorder and caregiver burden in spouses: the Nord-Trøndelag health study. *BMC Public Health*, *10*(1), 516. https://doi.org/10.1186/1471-2458-10-516
Jacob, G., & Bengel, J. (2000). Das Konstrukt Patientenzufriedenheit: Eine kritische Bestandsaufnahme. *Zeitschrift Für Klinische Psychologie, Psychiatrie Und Psychotherapie*, *48*, 280–301.
Jacobi, F., Höfler, M., Strehle, J., Mack, S., Gerschler, A., Scholl, L., … Wittchen, H.-U. (2014). Psychische Störungen in der Allgemeinbevölkerung. Studie zur Gesundheit Erwachsener in Deutschland und ihr Zusatzmodul Psychische Gesundheit (DEGS1-MH). *Der Nervenarzt*, *85*(1), 77–87. https://doi.org/10.1007/s00115-013-3961-y
Katsuki, F., Takeuchi, H., Konishi, M., Sasaki, M., Murase, Y., Naito, A., … Furukawa, T. A. (2011). Pre-post changes in psychosocial functioning among relatives of patients with depressive disorders after Brief Multifamily Psychoeducation: A pilot study. *BMC Psychiatry*, *11*. https://doi.org/10.1186/1471-244X-11-56
Kelders, S. M., Bohlmeijer, E. T., Pots, W. T. M., & van Gemert-Pijnen, J. E. W. C. (2015). Comparing human and automated support for depression: Fractional factorial randomized controlled trial. *Behaviour Research and Therapy*, *72*, 72–80. https://doi.org/10.1016/j.brat.2015.06.014
Kessler, R. C., Andrews, G., Colpe, L. J., Hiripi, E., Mroczek, D. K., Normand, S.-L. T., … Zaslavsky, A. M. (2002). Short screening scales to monitor population prevalences and trends in non-specific psychological distress. *Psychological Medicine*, *32*(6), 959–976. https://doi.org/10.1017/S0033291702006074
Klaghofer, R., Brähler, E. (2001). Konstruktion und Teststatistische Prüfung einer Kurzform der SCL-90–R [Construction and test statistical evaluation of a short version of the SCL-90–R]. *Zeitschrift Für Klinische Psychologie, Psychiatrie Und Psychotherapie*, *49(2)*, 115–124.
König, H.-H., Luppa, M., & Riedel-Heller, S. (2010). Die Kosten der Depression und die Wirtschaftlichkeit ihrer Behandlung. *Psychiatrische Praxis*, *37*(05), 213–215. https://doi.org/10.1055/s-0030-1248510
Laux, L., Glanzmann, P., Schaffner, P., Spielberger, C. D. (1981). *Das State-Trait-Angstinventar*. Göttingen: Beltz Test GmbH.
Leithäuser, T., & Volmerg, B. (1988). *Psychoanalyse in der Sozialforschung*. Wiesbaden: VS Verlag für Sozialwissenschaften. https://doi.org/10.1007/978-3-663-07773-2
Löwe, B., Spitzer, R.L., Zipfel, S., Herzog, W. (2002). *Gesundheitsfragebogen für Patienten (PHQ D). Komplettversion und Kurzform. Testmappe mit Manual, Fragebögen, Schablonen* (2nd ed.). Karlsruhe: Pfitzer.
Meister, R., Jansen, A., Berger, M., Baumeister, H., Bschor, T., Harfst, T., … Härter, M. (2018). Psychotherapie depressiver Störungen: Verfahren, Evidenz und Perspektiven. *Der Nervenarzt*,

89(3), 241–251. https://doi.org/10.1007/s00115-018-0484-6
Perlick, D. A., Rosenheck, R. R., Clarkin, J. F., Raue, P., & Sirey, J. (2001). Impact of family burden and patient symptom status on clinical outcome in bipolar affective disorder. *The Journal of Nervous and Mental Disease*, *189*(1), 31–37.
Petrowski, K., Schmalbach, B., Kliem, S., Hinz, A., & Brähler, E. (2019). Symptom-Checklist-K-9: Norm values and factorial structure in a representative German sample. *PLOS ONE*, *14*(4), e0213490. https://doi.org/10.1371/journal.pone.0213490
Polverejan, E., & Dragalin, V. (2020). Aligning treatment policy estimands and estimators—a simulation study in Alzheimer's disease. *Statistics in Biopharmaceutical Research*, *12*(2), 142–154. https://doi.org/10.1080/19466315.2019.1689845
Robert Koch-Institut. (2018). Gesundheitsberichterstattung des Bundes - Verlorene Erwerbstätigkeitsjahre in 1.000 Jahren für Deutschland. Retrieved from http://www.gbe-bund.de/oowa921-install/servlet/oowa/aw92/WS0100/_XWD_PROC?_XWD_374/2/XWD_CUBE.DRILL/_XWD_402/D.946/14321
Rummel-Kluge, C., Kluge, M., & Kissling, W. (2015). Psychoedukation bei Depression: Ergebnisse zweier Umfragen im Abstand von fünf Jahren in Deutschland, Österreich und der Schweiz. *Psychiatrische Praxis*, *42*(6), 309–312. https://doi.org/10.1055/s-0035-1552662
Schulz, K. F., Altman, D. G., & Moher, D. (2010). CONSORT 2010 Statement: updated guidelines for reporting parallel group randomised trials. *BMJ*, *340*, c332. https://doi.org/10.1136/bmj.c332
Shimazu, K., Shimodera, S., Mino, Y., Nishida, A., Kamimura, N., Sawada, K., … Inoue, S. (2011). Family psychoeducation for major depression: Randomised controlled trial. *British Journal of Psychiatry*, *198*(5), 385–390. https://doi.org/10.1192/bjp.bp.110.078626
Spitzer, R. L. (1999). Validation and Utility of a Self-report Version of PRIME-MD<SUBTITLE>The PHQ Primary Care Study</SUBTITLE>. *JAMA*, *282*(18), 1737. https://doi.org/10.1001/jama.282.18.1737
Steele, A., Maruyama, N., & Galynker, I. (2010). Psychiatric symptoms in caregivers of patients with bipolar disorder: A review. *Journal of Affective Disorders*, *121*(1–2), 10–21. https://doi.org/10.1016/j.jad.2009.04.020
van Wijngaarden, B., Schene, A. H., Koeter, M., Vázquez-Barquero, J. L., Knudsen, H. C., Lasalvia, A., & McCrone, P. (2000). Caregiving in schizophrenia: development, internal consistency and reliability of the Involvement Evaluation Questionnaire – European Version. *British Journal of Psychiatry*, *177*(S39), s21–s27. https://doi.org/10.1192/bjp.177.39.s21
van Winjgaarden, B. S. A. H. (1992). *The Involvement Evaluation Questionnaire*. Amsterdam: Department of Psychiatry, University of Amsterdam.
Vos, T., Flaxman, A. D., Naghavi, M., Lozano, R., Michaud, C., Ezzati, M., … Murray, C. J. L. (2012). Years lived with disability (YLDs) for 1160 sequelae of 289 diseases and injuries 1990-2010: A systematic analysis for the Global Burden of Disease Study 2010. *The Lancet*, *380*(9859), 2163–2196. https://doi.org/10.1016/S0140-6736(12)61729-2
WHO. (1998). *Wellbeing Measures in Primary Healh Care/The DEPCARE Project*. Stockholm.
Wiedemann, G., Rayki, O., Feinstein, E., & Hahlweg, K. (2002). The Family Questionnaire: Development and validation of a new self-report scale for assessing expressed emotion. *Psychiatry Research*, *109*(3), 265–279. https://doi.org/10.1016/S0165-1781(02)00023-9
Wittmund, B., Wilms, H.-U., Mory, C., & Angermeyer, M. C. (2002). Depressive disorders in spouses of mentally ill patients. *Social Psychiatry and Psychiatric Epidemiology*, *37*(4), 177–182. https://doi.org/10.1007/s001270200012
World Health Organization. (2017). *Depression and Other Common Mental Disorders: Global Health Estimates*. Geneva: World Health Organization.
