# Supplementary material for: An online program with individualized vs automated support for significant others of depressed individuals – study protocol of a randomized controlled trial
Source: BMC Psychiatry. 2022 Jul 28;22:511. doi: 10.1186/s12888-022-04035-6 (PMC9331487; doi:10.1186/s12888-022-04035-6)
Supplement: Supplementary file 2 — Additional file 2. Interview questions. [file 12888_2022_4035_MOESM2_ESM.docx]

**Interview Questions**

(English translation from the German original)

**Part 1 – General Experiences**

- Please tell us how you have been doing with the accompanied online program in the last weeks.

**Part 2 – Experiences with psychological support**

Individualized Support:

- During the four weeks in which you were using the online coach, you received regular e-mails from a psychological support person and had the opportunity to share your experiences with them and ask questions. Tell me how that was for you.
- What were your initial expectations of the psychologist? Were these expectations met?
- How did you experience the relationship with the psychologist?
- How did the exchange with the psychological support person influence your work with the online program?
- Could you take anything home from the exchange? (Which aspects of the exchange were important to you?)
- How was the setting for you, ie the online format, the duration of the support, the anonymity, the frequency of the messages?
- How did you experience the end of the e-mail support? [if problematic: do you have an idea what would have helped to make the end easier for you?]

Automated support:

- During the four weeks that you were using with the online coach, you received emails from the program three times a week. How was that for you?
- How helpful did you find the psychological support e-mails?
- How appropriate did the tips seem to you?

**Part 3 – Effects of the program**

- Now we come to the last part of the interview, where we are interested in the effects of the accompanied online program. Has anything changed for you as a result of participating in the program?
- Have you noticed changes in the way you deal with the depressive symptoms of your relative (partner, etc.)?
- Have you noticed any changes in your relationship with your relative (partner, etc.)?
- Has the way you deal with yourself during this difficult time changed, how do you take care of yourself?
- Has the knowledge content of the online program had an impact on your situation?

Follow-up questions (if not reported spontaneously):

- Overall, what was particularly helpful to you?
- Was there anything that was difficult or problematic?
- Is there anything that you missed in the online intervention?
- That would be all the questions from our side. Is there anything else important about the online intervention that we haven't discussed?
